# REDD1/DDIT4 counteracts endoplasmic reticulum stress-induced apoptosis by controlling the expression of death receptor TRAILR2/DR5 in cancer cells

**DOI:** 10.1038/s41419-026-08648-7

**Published:** 2026-03-28

**Authors:** Rocío Mora-Molina, Younes El Yousfi, Cathrin Hagenlocher, Francisco Javier Fernández-Farrán, Markus Rehm, Carmen Palacios, Maria A. Christophorou, Abelardo López-Rivas

**Affiliations:** 1 Centro Andaluz de Biología Molecular y Medicina Regenerativa-CABIMER, CSIC-Universidad de Sevilla-Universidad Pablo de Olavide, Avda Américo Vespucio 24, Sevilla, Spain; 2 University of Stuttgart, Institute of Cell Biology and Immunology, Stuttgart, Germany; 3 University of Stuttgart, Stuttgart Research Center Systems Biology, Stuttgart, Germany; 4 Present Address: Epigenetics, Babraham Institute, Cambridge, UK; 5 Present Address: University of Stuttgart, Institute of Biochemistry, Stuttgart, Germany

**Keywords:** Endoplasmic reticulum, Colon cancer

## Abstract

Regulated in development and DNA damage response-1 (REDD1/DDIT4) is induced in response to environmental stress to restrain the mechanistic target of rapamycin complex 1 (mTORC1) signaling as an adaptive strategy to restore cellular homeostasis. Interestingly, REDD1/DDIT4 expression is upregulated in several tumor types including colorectal cancer, suggesting it may have a role in tumourigenesis. Here, we report that activating transcription factor 4 (ATF4)-dependent REDD1/DDIT4 expression is required for survival of colon tumor cells undergoing endoplasmic reticulum (ER) stress through the modulation of TRAILR2/DR5 gene expression. Our findings further demonstrate that resistance to ER stress-induced apoptosis in multicellular tumor spheroids (MCTS) is associated with constitutive expression of REDD1/DDIT4 and diminished mTORC1 activity. CRISPR/Cas9-mediated deletion of REDD1/DDIT4 markedly increases TRAILR2/DR5 expression and enhances apoptosis in spheroids exposed to ER stress. Interestingly, RNA sequencing analysis reveals that the loss of the transcriptional regulator EVI-1/MECOM in cells deficient in REDD1/DDIT4 amplifies the ER stress-induced upregulation of TRAILR2/DR5, leading to enhanced apoptosis. In summary, our findings underscore the crucial role of REDD1/DDIT4 in regulating TRAILR2/DR5-induced caspase-8 activation and apoptosis under chronic ER stress, by inhibiting mTORC1 activity and promoting EVI-1/MECOM-mediated suppression of TRAILR2/DR5 gene expression.

## Introduction

The tumor microenvironment is characterized by severe hypoxia, nutrient deprivation and acidosis that provoke the accumulation of unfolded and misfolded proteins in the endoplasmic reticulum (ER) [1, 2]. This results in ER stress and the activation of an adaptive response to promote tumor survival and progression [1, 3]. Protein sensors located in the luminal face of the ER membrane activate an intracellular signaling mechanism called the unfolded protein response (UPR) [4]. Activation of these signaling pathways leads to a reduction in the influx of proteins into the ER, triggers protein degradation pathways and increases the folding capacity of the ER [5]. However, above a certain threshold, intense or unresolved ER stress results in cell death by apoptosis [6]. Up-regulation of pro-apoptotic proteins and down-regulation of anti-apoptotic proteins have been observed in cells undergoing apoptosis upon ER stress [7–13]. Up-regulation of TRAIL receptor 2/Death Receptor 5 (TRAILR2/DR5) expression and its intracellular activation in a TRAIL-independent manner mediates the execution of a caspase-8-dependent apoptotic pathway upon ER stress in both, tumor and oncogenically transformed cells [8, 9, 11, 13]. Moreover, it has been reported that misfolded proteins directly bind to and activate TRAILR2/DR5 in the ER-Golgi intermediate compartment to induce caspase-8 activation and apoptosis [14].

In response to ER stress, regulated in development and DNA damage response-1 (REDD1/DDIT4) protein is up-regulated, leading to the inhibition of the mammalian target of rapamycin complex 1 (mTORC1) in a tuberous sclerosis complex (TSC1/TSC2)-dependent manner [15]. Furthermore, loss of the upstream regulators of the mTOR pathway TSC1 and TSC2 leads to constitutive activation of mTORC1 and tumor development [16–18]. Interestingly, tumor cells harboring an activated mTOR pathway are more sensitive to ER stress-induced cell death [19, 20], although the molecular mechanism underlying this cell death process remains poorly understood. In addition to its role in controlling mTORC1 signaling, REDD1/DDIT4 also has mTORC1-independent functions. These include preventing mitochondrial ROS production and stabilizing HIF1α [21], inducing autophagy [22], or activating proinflammatory signaling pathways [23]. Although a tumor suppressor role has been reported for REDD1/DDIT4 in some malignancies [24–26], in colorectal cancer the elevated expression of REDD1/DDIT4 may have a pro-tumoural function promoting survival of stressed tumor cells [27]. Despite this observation, how REDD1 modulates colon tumor cells' response to ER stress remains poorly understood.

EVI-1/MECOM is a zinc finger transcription factor commonly linked to oncogenesis and apoptosis [28–30]. Additionally, EVI-1/MECOM can interact with the corepressor proteins C-terminal Binding Protein (CtBP) via a repressive domain located between two zinc finger domains in its C-terminal region [31–33]. Notably, CtBPs are known to play a key role in repressing the expression of pro-apoptotic genes [34, 35], including TRAILR1/DR4 and TRAILR2/DR5 [36], in tumor cells. In this study, we show that REDD1/DDIT4 plays a critical role in preventing apoptosis activation in response to chronic ER stress in colon cancer cells. This is accomplished by inhibiting mTORC1 and regulating TRAILR2/DR5 expression through EVI-1/MECOM-dependent transcriptional repression, with CtBP proteins providing additional regulation in certain cellular contexts. Our findings suggest that elevated REDD1/DDIT4 expression in colorectal cancer may confer a growth advantage in the hostile tumor microenvironment by delaying ER stress-induced upregulation of TRAILR2/DR5 and the activation of caspase-8-dependent apoptosis, thereby facilitating tumor growth and progression.

## Results

### ATF4-dependent REDD1/DDIT4 expression is required for survival of colon cancer cells undergoing ER stress

Different stressful conditions of the tumor microenvironment cause ER stress and trigger the UPR [1]. Although a tumor-suppressor role has been proposed for REDD1/DDIT4 in some contexts [21, 25], in colorectal cancer REDD1/DDIT4 induction facilitates cellular adaptation in hypoxic conditions [27], and promotes the tumor-supportive function of tumor-associated macrophages (TAMs) [37]. However, despite the observation that REDD1/DDIT4 is frequently upregulated in colorectal cancer [27], its function in the response of colon tumor cells to ER stress has not been elucidated.

**Fig. 1:**
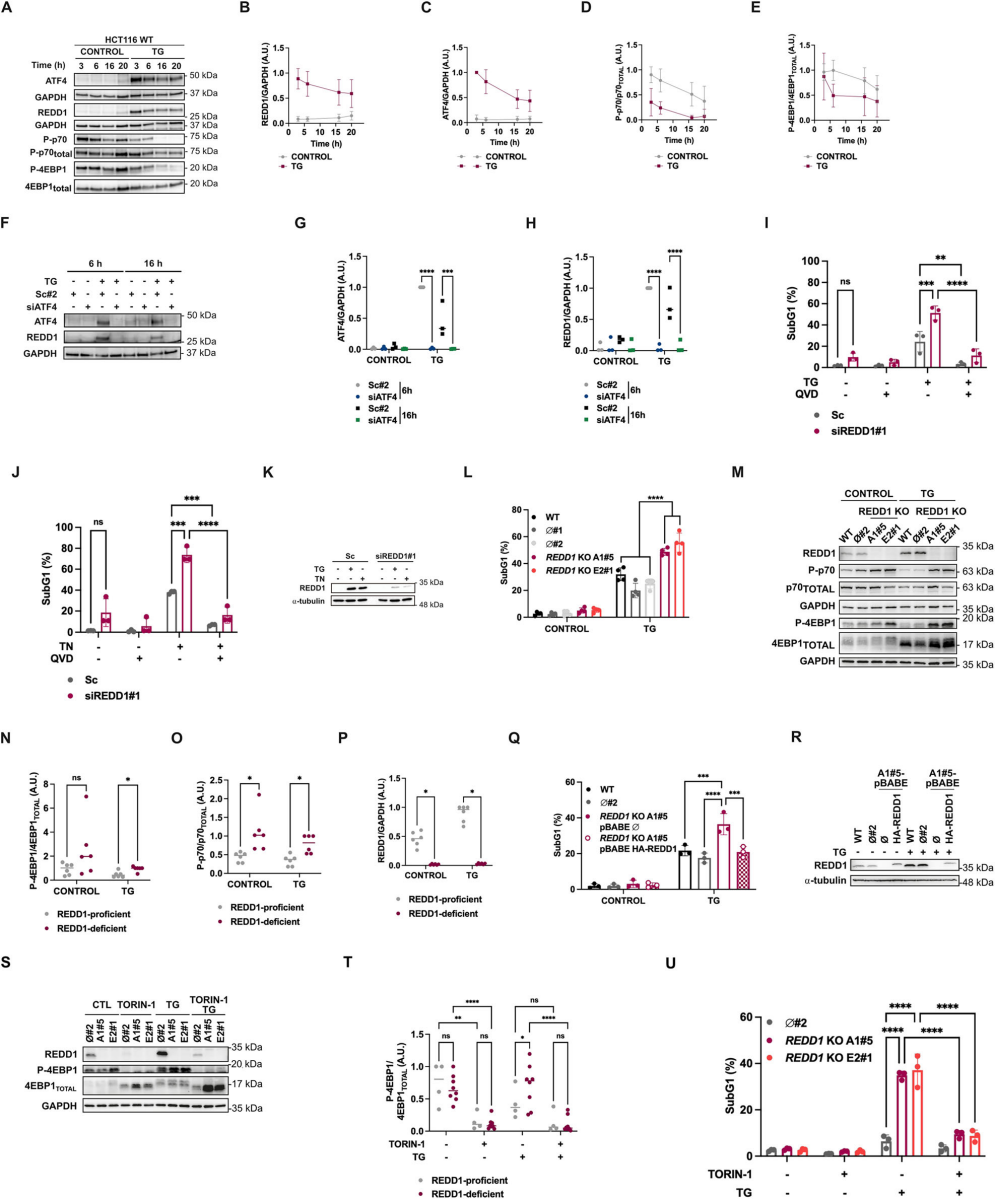
Inhibition of ATF4-Induced REDD1/DDIT4 expression during ER Stress sensitizes HCT116 colon cancer cells to apoptosis. **A** HCT116 cells were treated with thapsigargin (TG, 100 nM) for the indicated times. REDD1 levels and phosphorylation of 4EBP1 and p70S6K, downstream targets of mTORC1, were assessed by western blotting. GAPDH was used as loading control. Representative blot from five independent experiments. **B–E** Quantification of western blots from (**A**): REDD1 (**B**) and ATF4 (**C**) levels were normalized to loading controls, and phospho-p70S6K (**D**) and phospho-4EBP1 (**E**) to their total protein levels. **F** HCT116 cells were transfected with either scrambled oligonucleotide#2 (Sc#2) or ATF4 siRNA (siATF4) for 48 h, followed by TG treatment (100 nM) for the indicated times. ATF4 knockdown and REDD1 induction were assessed in whole cell extracts by western blotting. GAPDH was used as a loading control. **G**,**H** Quantification of western blots from (**F**): ATF4 (**G**) and REDD1 (**H**) levels were normalized to the loading control (*n* = 3. ***p ≤ 0.001; ****p ≤ 0.0001; two-way ANOVA with Tukey’s multiple comparisons test). **I**,**J** HCT116 cells were transfected with scrambled oligonucleotide (Sc) or REDD1 siRNA (siREDD1#1) for 48 h, followed by treatment with or without TG (100 nM) for 24 h (**I**) or tunicamycin (TN, 1 μg/mL) for 48 h (**J**) in the presence or absence of the pan-caspase inhibitor Q-VD-OPh (QVD, 20 μM). Apoptosis was quantified by subG1 analysis (*n* = 3. ns = not statistically significant; **p ≤ 0.01; ***p ≤ 0.001; ****p ≤ 0.0001; two-way ANOVA with Tukey’s multiple comparisons test). **K** REDD1 knockdown from (**I**,**J**) was confirmed by western blotting in whole-cell extracts, with α-tubulin as a loading control. **L** HCT116 WT cells, ø#1 and ø#2 clones, as control cells, and *REDD1 KO* A1#5 and E2#1 clones were treated or not with TG (100 nM) for 24 h. Apoptosis was determined after treatment by subG1 analysis (*n* = 3. ****p ≤ 0.0001. Two-way ANOVA. Tukey’s multiple comparisons test). **M** HCT116 WT, ø#2, *REDD1* KO A1#5 and *REDD1* KO E2#1 were treated or not with TG (100 nM) for 24 h. REDD1 and phosphorylation of 4EBP1 and p70S6K were determined in whole cell extracts by western blotting. GAPDH was used as protein-loading control. **N**,**P** Quantification of western blots from (**M**): phospho-4EBP1 (**N**) and phospho-p70S6K (**O**) were normalized to their total levels, and REDD1 (**P**) to the loading control (*n* = 3. WT and Ø#2 (REDD1-proficient) data were pooled for analysis; *REDD1/DDIT4* KO A1#5 and E2#1 (REDD1-deficient) data were pooled for analysis. ns = not statistically significant; *p ≤ 0.05. Multiple unpaired t test). **Q** HCT116 WT cells and ø#2 clone, as control cells, and *REDD1/DDIT4 KO* A1#5 clone carrying pBABE-ø or pBABE-HA-REDD1 vectors were treated or not with TG (100 nM) for 24 h. Apoptosis was determined after treatment by subG1 analysis (*n* = 3. ***p ≤ 0.001; ****p ≤ 0.0001. Two-way ANOVA. Tukey’s multiple comparisons test). **R** REDD1 levels from **Q** were assessed in whole cell extracts by western blotting. α–tubulin was used as protein-loading control. **S** HCT116 ø#2 control clone and *REDD1/DDIT4* KO A1#5 and E2#1 clones were treated with or without Torin-1 (250 nM) for 2 h, followed by the addition of TG (100 nM) for a further 16 h-period. REDD1 and phosphorylation of 4EBP1 were determined in whole cell extracts by western blotting. GAPDH was used as protein-loading control. **T** Quantification of western blots from (**S**): Phospho-4EBP1 was normalized to its total levels (*n* = 4. *REDD1/DDIT4* KO A1#5 and E2#1 (REDD1-deficient) data were pooled for analysis. ns = not statistically significant; **p ≤ 0.01; ***p ≤ 0.001; ****p ≤ 0.0001; two-way ANOVA with Tukey’s multiple comparisons test). **U** HCT116 ø#2 control clone and *REDD1* KO A1#5 and E2#1 clones were treated with or without Torin-1 (250 nM) for 2 h, followed by the addition of TG (100 nM) for a further 24 h-period. After treatment, apoptosis was assessed by subG1 analysis (*n* = 3. ****p ≤ 0.0001; Two-way ANOVA. Tukey’s multiple comparisons test).

To investigate this issue, we first evaluated REDD1/DDIT4 expression in the HCT116 human colon cancer cell line following exposure to ER stress-inducing agents. Treatment with the ER stress inducer thapsigargin (TG) resulted in an upregulation of REDD1/DDIT4 protein expression, which was detectable as early as 3 hours after TG was added to HCT116 cell cultures (Fig. 1A, B). The increase in REDD1/DDIT4 expression was associated with the transcription factor ATF4 (Fig. 1A, C), a well-known mediator of the PERK branch of the UPR [38], and inhibition of the kinase activity of the mTORC1 complex, as indicated by 4EBP1 and p70S6K phosphorylations, substrates of the mTORC1 kinase (Fig. 1A, D, E). Notably, ATF4 expression decreases over the course of TG treatment. This reflects attenuation of the PERK pathway that occurs in the late phases of UPR as a result of a negative feedback loop [39, 40], which, under conditions of unmitigated ER stress, promotes proteotoxicity and cell death over survival [41].

To further investigate the mechanism underlying the observed elevation of REDD1/DDIT4 levels upon ER stress, we determined the role of ATF4, which has been previously associated with REDD1/DDIT4 transcriptional regulation [42–44]. In fact, as shown in Fig. 1A–C, REDD1/DDIT4 protein expression exhibited the same temporal pattern as ATF4. More importantly, the knockdown of ATF4 expression by siRNA interference markedly inhibited REDD1/DDIT4 increase in HCT116 cells treated with TG (Fig. 1F–H), confirming data obtained in other cell types [42–44].

To elucidate the role of REDD1/DDIT4 in the apoptotic response of colon tumor cells to ER stress, we assessed the susceptibility of control and REDD1/DDIT4 knockdown HCT116 cells to the ER stress inducers TG and tunicamycin (TN). REDD1/DDIT4 knockdown resulted in a significant increase in the apoptotic population following treatment with either TG or TN (Fig. 1I–K) an effect that was substantially inhibited by co-treatment with the pan-caspase inhibitor Q-VD-Oph. To confirm these findings, we used two additional siRNA sequences targeting REDD1/DDIT4 prior to exposure to the ER stress inducers. All sequences significantly enhanced apoptosis following treatment with TG (Fig. S1A) or TN (Fig. S1B, C). Moreover, after ER stress induction, REDD1/DDIT4 knockdown cells exhibited sustained mTORC1 kinase activity, as demonstrated by the phosphorylation of its downstream targets, 4EBP1 and p70S6K (Fig. S1D–K). These findings were not restricted to the HCT116 cell line, as the colorectal cancer cell line HT29 also exhibited a significant sensitization to TG-induced apoptosis upon REDD1 depletion (Fig. S1L–N). To characterize the role of REDD1/DDIT4 in HCT116 colon cancer cells undergoing ER stress-induced apoptosis, *REDD1/DDIT4* knockout (KO) cells were generated using CRISPR/Cas9 gene editing. As shown in Fig. 1L, *REDD1/DDIT4* KO clones exhibited heightened sensitivity to TG treatment compared to parental cells or REDD1/DDIT4-expressing control clones, validating the data obtained with siRNAs. Consistent with REDD1/DDIT4’s canonical function in regulating mTORC1 activity by inhibiting Akt-mediated control of the TSC [15], *REDD1/DDIT4* KO cells exhibited enhanced mTORC1 activation, both under control and treatment conditions, as assessed by the phosphorylation of 4EBP1 and p70S6K (Fig. 1M–P). To confirm that the increased apoptosis observed upon ER stress in *REDD1/DDIT4* KO cells was specifically due to the loss of REDD1/DDIT4, we restored its expression by infecting these KO cells with retroviruses carrying the HA-REDD1/DDIT4 expression vector. Notably, the enhanced sensitivity to TG-induced apoptosis was significantly reduced in *REDD1/DDIT4* KO clones ectopically expressing HA-REDD1/DDIT4 (Fig. 1Q–R and Fig. S1O, P). This resulted in apoptosis levels comparable to those observed in parental cells or in a REDD1/DDIT4-expressing control clone treated with TG.

To evaluate the role of mTOR in REDD1/DDIT4-mediated sensitization to ER stress, REDD1/DDIT4-expressing and *REDD1/DDIT4* KO clones were pre-treated for 2 h with the mTORC1/2 inhibitor Torin-1 before being treated with TG for an additional 24 h. As expected, Torin-1 completely inhibited phosphorylation of 4EBP1 and, therefore mTORC1 activity (Fig. 1S, T), which prevented TG-induced apoptosis in REDD1/DDIT4-proficient and deficient cells (Fig. 1U). Thus, maintained mTORC1 signaling promotes ER-stress sensitization in the absence of REDD1. Additionally, Torin-1 pre-treatment exhibited a similar effect in HT29 cancer cells following downregulation of REDD1/DDIT4 (Fig. S1Q–S). Interestingly, higher, although non-significant, apoptosis levels were observed in REDD1/DDIT4-deficient cells after mTORC1 inhibition (Fig. 1U), which could indicate that REDD1 may also operate through mTORC1-independent functions.

### REDD1/DDIT4 plays a key role in the control of ER stress-induced activation of TRAILR2/DR5-mediated apoptosis

ER stress-induced activation of the UPR triggers signaling pathways aimed at restoring ER proteostasis to promote cell survival [5, 45]. However, unmitigated ER stress shifts the cellular response from adaptation to apoptotic cell death, in which either the intrinsic [6, 12, 46], extrinsic [8, 9, 14], or both apoptotic pathways [7] have been implicated. In the HCT116 model, activation of the PERK/ATF4/CHOP branch of the UPR under chronic ER stress conditions results in upregulation of TRAILR2/DR5 expression, leading to the formation of an intracellular DISC and activation of the extrinsic apoptosis pathway, independent of the TRAIL ligand [8, 14].

**Fig. 2:**
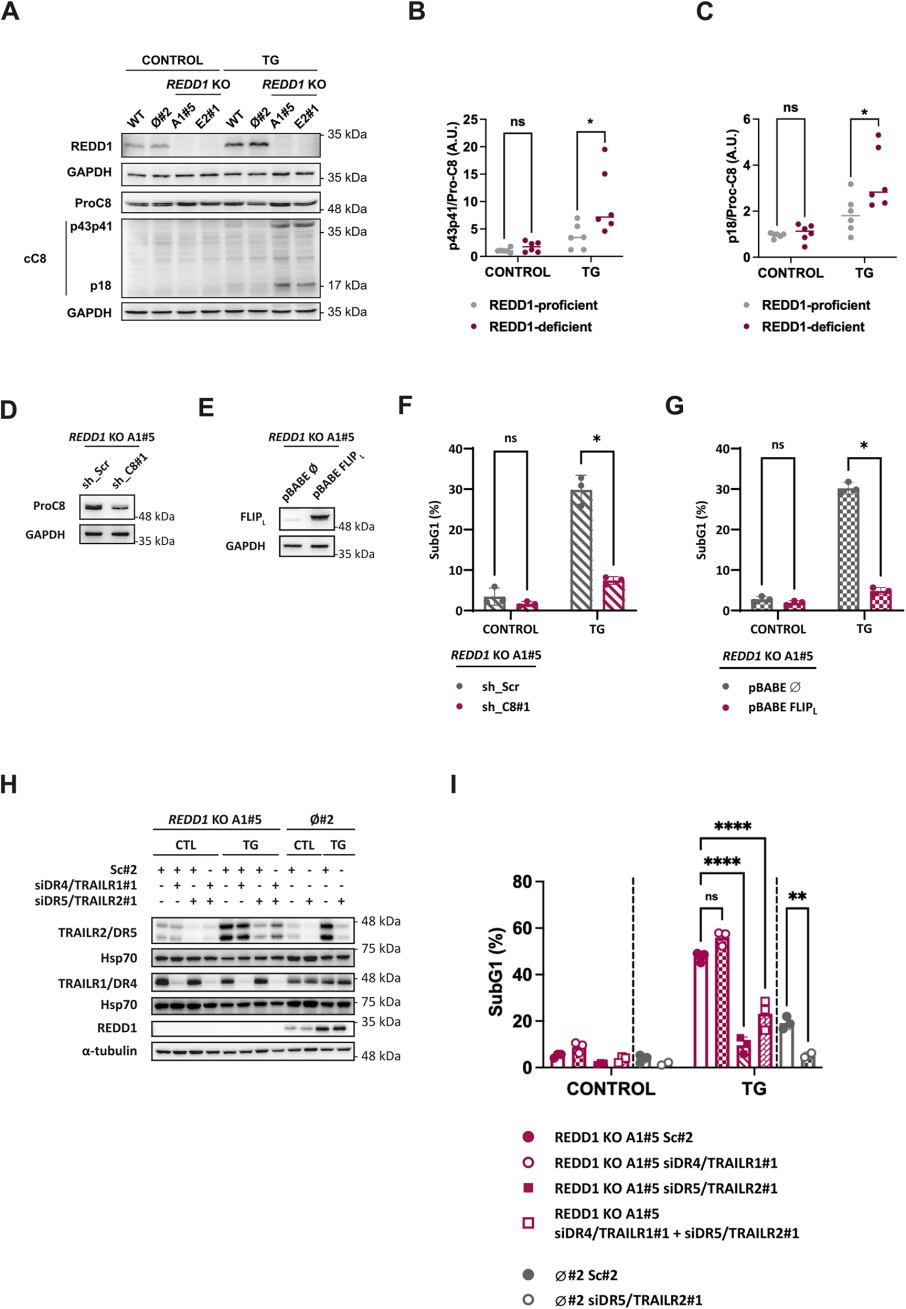
The extrinsic apoptotic pathway drives ER stress-induced apoptosis in REDD1/DDIT4 knockout cells. **A** HCT116 WT, ø#2, *REDD1/DDIT4* KO A1#5 and E2#1 cells were treated or not with TG (100 nM) for 24 h. REDD1, procaspase-8 (ProC8) and cleaved caspase-8 (cC8) levels were determined in whole-cell extracts by western blotting. GAPDH was used as protein-loading control. **B**,**C** Quantification of western blots from (**A**)**:** p43p41 (**B**) and p18 (**C**) fragments were normalized to procaspase-8 (*n* = 3. WT and Ø#2 (REDD1-proficient) data were pooled for analysis; *REDD1/DDIT4* KO A1#5 and E2#1 (REDD1-deficient) data were pooled for analysis. ns = not statistically significant; *p ≤ 0.05. Multiple unpaired t test). **D** Western blot of HCT116 *REDD1/DDIT4* KO A1#5 sh_Scrambled (sh_Scr) or sh_Caspase-8#1 (sh_C8#1) cells. Caspase-8 knockdown was determined in whole cell extracts by western blotting. GAPDH was used as protein-loading control. **E** Western blot of HCT116 *REDD1/DDIT4* KO A1#5 pBABE-ø or pBABE-FLIP_L_ cells. FLIP_L_ overexpression was determined in whole cell extracts by western blotting. GAPDH was used as protein-loading control. **F**,**G** HCT116 *REDD1/DDIT4* KO sh_Scr, sh_C8#1 cells (**F**) or HCT116 *REDD1* KO A1#5 pBABE-ø or pBABE-FLIP_L_ cells (**G**) were treated or not with TG (100 nM) for 24 h. Apoptosis was determined after treatment by subG1 analysis (*n* = 3. ns = not statistically significant; *p ≤ 0.05. Multiple unpaired t test). **H**,**I** HCT116 *REDD1/DDIT4* KO A1#5 cells were transfected with scrambled oligonucleotide#2 (Sc#2), siTRAILR1/DR4#1, and/or siTRAILR2/DR5#1, as indicated in the figure. After 48 h, the cells were treated with TG (100 nM) for an additional 16 h (**H**) or 24 h (**I**) period. In parallel, HCT116 ø#2 cells were transfected with scrambled oligonucleotide#2 (Sc#2) or siTRAILR2/DR5#1 to serve as a control for the role of TRAILR2/DR5 in apoptosis induction in REDD1-expressing cells during ER stress. **H** TRAILR1/DR4 and TRAILR2/DR5 knockdowns and REDD1 induction were confirmed by western blot analysis. Hsp70 and α-tubulin were used as loading controls for protein normalization. **I** Apoptosis was determined after treatment by subG1 analysis (*n* = 3. ns = not statistically significant; *p ≤ 0.05; Two-way ANOVA. Tukey’s multiple comparisons test).

Given the role of the extrinsic apoptotic pathway triggered by TRAIL receptors in ER stress-induced cell death in HCT116 tumor cells [8, 11], we hypothesized that the increased susceptibility of REDD1/DDIT4-deficient tumor cells to ER stress could be attributed to enhanced activation of caspase-8, a key caspase in the execution of the extrinsic apoptotic pathway. To test this, we assessed caspase-8 activation by examining its protein processing following TG treatment. As shown in Fig. 2A–C, REDD1/DDIT4-deficient cells exhibited significantly increased caspase-8 processing and subsequent activation compared to control cells. To further explore the involvement of the extrinsic apoptotic pathway in the heightened sensitivity of *REDD1/DDIT4* KO cells to ER stress, we inhibited caspase-8 activation either by caspase-8 knockdown (Fig. 2D) or by overexpressing FLIP_L_ (Fig. 2E), which is known to prevent caspase-8 processing [11, 47, 48]. As shown in Fig. 2F, G, the increased susceptibility of *REDD1/DDIT4* KO cells to TG treatment was significantly reduced in cells with caspase-8 knockdown or FLIP_L_ overexpression. We also explored the role of pro-apoptotic TRAIL receptors, TRAILR1/DR4 and TRAILR2/DR5, in the increased susceptibility of REDD1/DDIT4-deficient cells to ER stress through siRNA-mediated knockdown. TRAILR1/DR4 and/or TRAILR5/DR5 knockdowns wereconfirmed by western blot (Fig. 2H). Notably, only knockdown of TRAILR2/DR5 significantly prevented ER stress-induced apoptosis in the absence of REDD1/DDIT4 (Fig. 2I). Taken together, these results suggest that REDD1/DDIT4 plays a role in delaying excessive caspase-8 activation and apoptosis via the extrinsic pathway, thereby promoting cell adaptation rather than cell death.

### Multicellular tumor spheroids (MCTSs) derived from REDD1/DDIT4 knockout (KO) cells exhibit increased susceptibility to apoptosis induced by ER stress

**Fig. 3:**
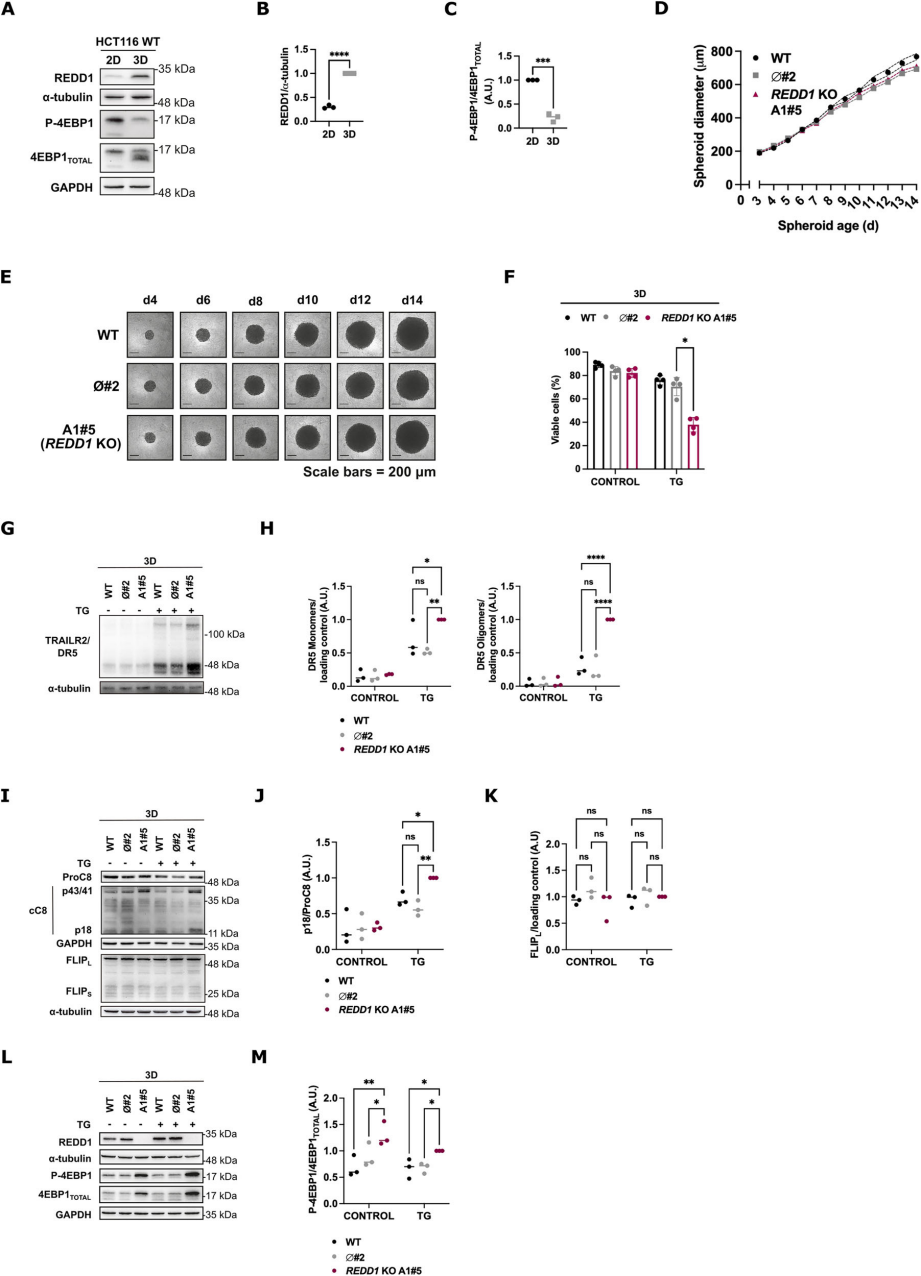
Enhanced upregulation of TRAILR2/DR5 and increased sensitivity to apoptosis under ER stress in multicellular tumor spheroids derived from REDD1/DDIT4 knockout cells. **A** REDD1/DDIT4 levels and 4EBP1 phosphorylation in cultures of HCT116 cells growing in 2D or as spheroids (3D) for 10 days. REDD1 levels and 4EBP1 phosphorylation were analyzed in whole cell extracts by western blotting. **B**,**C** Western blot quantification from (**A**): REDD1 (**B**) levels normalized to the loading control, and phospho-4EBP1 (**C**) to its total protein levels (*n* = 3. ***p ≤ 0.001; ****p ≤ 0.0001; unpaired t test). **D**,**E** MCTSs were cultivated in agarose-coated 96-well plates with the media changed every third day. Images were captured using a Leica inverted digital microscope, and the diameter was measured with ImageJ software as described in the Materials and Methods. **D** Graph represents mean values ± SEM (dashed lines) of one experiment (6 spheroids per cell line). **E** Pictures are representative of the experiment. Scale bars = 200 μm. **F** 10-day-old spheroids of HCT116 WT cells, ø#2 and *REDD1/DDIT4* KO A1#5 clones were treated with TG (100 nM) for 3 days and cell viability was analyzed by flow cytometry after staining with Annexin V-FITC and propidium iodide (PI) (*n* = 4. ****p ≤ 0.0001; Two-way ANOVA. Tukey’s multiple comparisons test). **G–M** Western blot images and quantification of HCT116 WT cells, ø#2 and *REDD1/DDIT4* KO A1#5-derived spheroids after 1 day of TG (100 nM) treatment. α-tubulin and GAPDH were used as protein-loading controls. Representative blot of three independent experiments. ns = not statistically significant; *p ≤ 0.05; **p ≤ 0.01; ****p ≤ 0.0001; two-way ANOVA with Tukey’s multiple comparisons test. TRAILR2/DR5 levels (**G**) and normalization (**H**) to the loading control. ProCaspase-8 (ProC8), cleaved caspase-8 (cC8) and FLIP_L_ levels (**I**). p18 fragment normalized to ProCaspase-8 (**J**) and FLIP_L_ normalized to the loading control (**K**). REDD1 levels and phosphorylation of 4EBP1 (**L**). Phospho-4EBP1 normalized to its total protein levels (**M**).

MCTSs, greater than 500 μm in size, more accurately replicate the three-dimensional architecture of growing tumors compared to traditional 2D cell cultures [49–51]. Previously, we reported that 3D spheroids derived from HCT116 tumor cells display resistance to ER stress-induced apoptosis compared to their 2D counterparts [11]. Given that REDD1/DDIT4 is a stress-induced protein, and hypoxia and nutrient starvation markers can be observed in HCT116-derived spheroids of this size compared to 2D cell cultures [52], we hypothesized that REDD1/DDIT4 levels might be upregulated in spheroid cultures. Indeed, analysis of REDD1/DDIT4 protein expression in whole cell extracts revealed that its levels were significantly higher in spheroids than in 2D cultures of HCT116 cells (Fig. 3A, B). Moreover, this upregulation of REDD1/DDIT4 was associated with reduced mTORC1 activity, as indicated by decreased phosphorylation of its downstream target 4EBP1 (Fig. 3A, C).

Next, we investigated whether depleting REDD1/DDIT4 in HCT116 cells would enhance the sensitivity of MCTSs to apoptosis induced by TG. Spheroids were generated from both control and *REDD1/DDIT4* KO cells, and after confirming that the loss of REDD1/DDIT4 expression did not affect spheroid growth (Fig. 3D, E), we assessed their response to ER stress-induced apoptosis. As shown in Fig. 3F, spheroids lacking REDD1/DDIT4 were markedly more susceptible to TG-induced apoptosis compared to spheroids derived from either the parental cells or a control REDD1/DDIT4-expressing clone. Notably, the increased apoptosis observed in *REDD1/DDIT4* KO spheroids was accompanied by elevated levels of TRAILR2/DR5 protein (Fig. 3G, H) and caspase-8 activation following TG treatment without affecting FLIP_L_ levels (Fig. 3I–K). Interestingly, despite TG treatment, the phosphorylation of 4EBP1—and thus mTORC1 activity—was significantly higher in *REDD1/DDIT4* KO spheroids, confirming that REDD1/DDIT4 regulates mTORC1 activity in MCTSs (Fig. 3L, M). In summary, these findings suggest that REDD1/DDIT4 plays a protective role in colon tumor cells under ER stress by delaying the upregulation of TRAILR2/DR5, thereby preventing premature activation of caspase-8 and the initiation of the extrinsic apoptotic pathway.

### REDD1/DDIT4 regulates TRAILR2/DR5 expression in colon cancer cells under ER stress

**Fig. 4:**
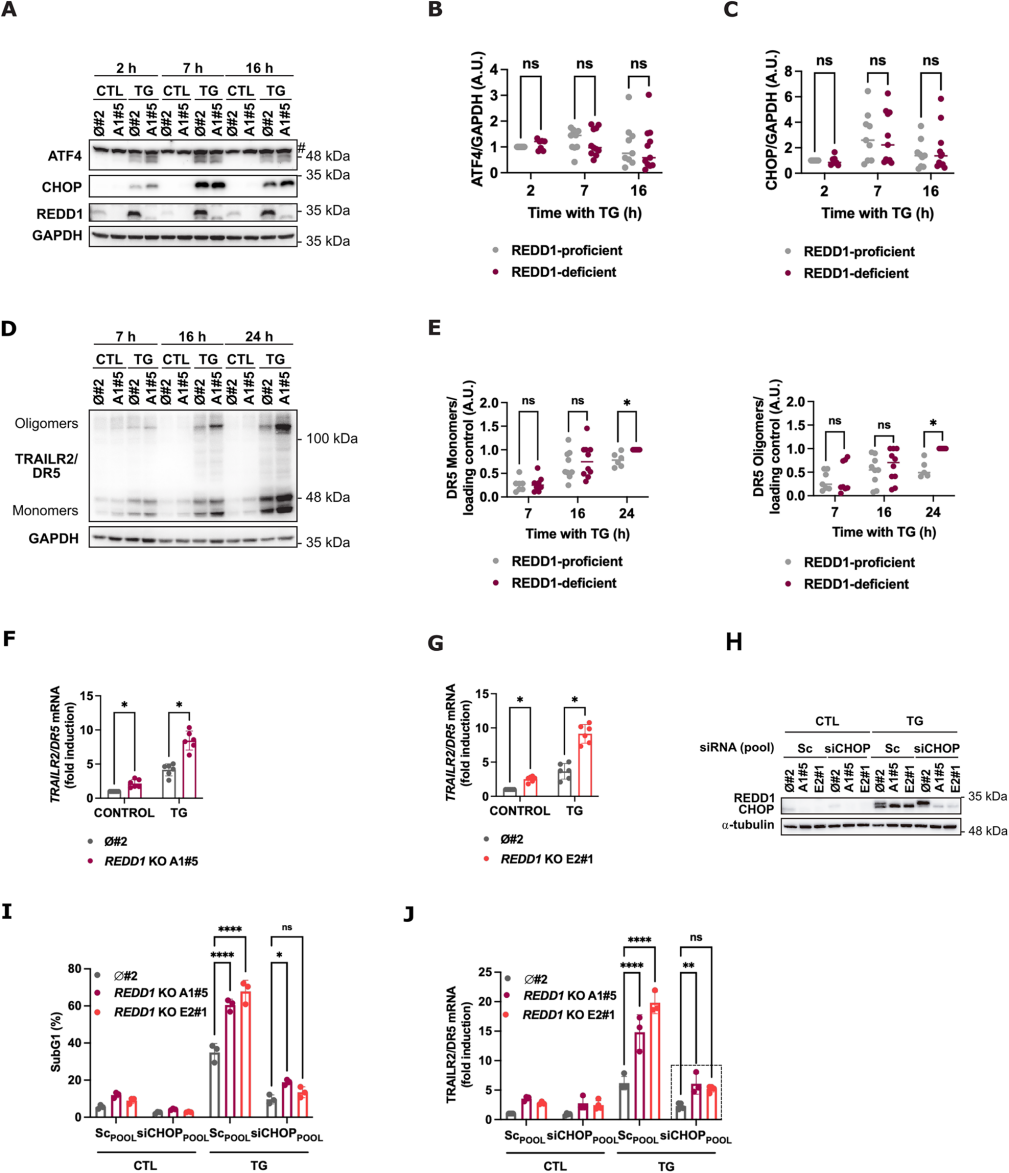
Similar activation of the PERK pathway and differential upregulation of TRAILR2/DR5 in response to ER stress between control and REDD1/DDIT4 knockout cells. HCT116 control clone (ø#2) and *REDD1/DDIT4* KO A1#5 were treated with TG (100 nM) for the indicated times (**A–F)**. **A** Induction of ATF4 and CHOP proteins and REDD1 deletion were determined in whole cell extracts by western blotting. GAPDH was used as protein-loading control. #: unspecific band. **B**,**C** Quantification of western blots from (**A**): ATF4 (**B**) and CHOP (**C**) were normalized to the loading control (*n* = 7 (2 h) or 9 (7 h, 16 h). ns not statistically significant, *p ≤ 0.05. Multiple unpaired t test (**D**) TRAILR2/DR5 protein levels were determined in whole-cell extracts by western blotting. GAPDH was used as protein-loading control. **E** Quantification of western blots from (**D**): TRAILR2/DR5 monomers and oligomers were normalized to the loading control (*n* = 6 (7 h, 24 h) or 9 (16 h). ns not statistically significant, *p ≤ 0.05. Multiple unpaired t test. **F**,**G** ø#2 control and *REDD1/DDIT4* KO A1#5 (**F**) and E2#1 (**G**) clones were treated with TG (100 nM) for 7 h. *TRAILR2/DR5* mRNA relative levels in control and *REDD1/DDIT4* KO clones were examined by RT-qPCR. (*n* = 6. *p ≤ 0.05; Multiple unpaired t test). **H–J** Cells from HCT116 control clone ø#2 and *REDD1/DDIT4* KO clones (A1#5 and E2#1) were transfected with a pool of scrambled oligonucleotides (Sc_POOL_) or CHOP (CHOP_POOL_), as indicated in the figure, for 48 h prior to TG (100 nM) treatment. **H** CHOP knockdown and REDD1 induction were assessed in whole cell extracts by western blotting after 16 h of TG treatment. α-tubulin was used as protein-loading control. **I** Apoptosis was determined after 24 h of TG treatment by subG1 analysis (*n* = 3. ns not statistically significant; ****p ≤ 0.0001; *p ≤ 0.05.; Two-way ANOVA. Tukey’s multiple comparisons test). **J** TRAILR2/DR5 expression was assessed by RT-qPCR after 16 h of treatment with TG (*n* = 3. ns not statistically significant; ****p ≤ 0.0001; *p ≤ 0.05; Two-way ANOVA. Tukey’s multiple comparisons test).

In response to persistent ER stress, the PERK branch of the UPR can activate the extrinsic apoptotic pathway by both downregulating FLIP levels [11] and upregulating TRAILR2/DR5 expression via CHOP induction [8, 9, 13]. Given the critical role of the PERK pathway in regulating TRAILR2/DR5 expression and, consequently, ER stress-induced apoptosis, we first investigated whether the loss of REDD1/DDIT4 would enhance PERK pathway activation. However, analysis of PERK pathway activation, assessed by measuring ATF4 and CHOP protein levels after TG treatment, revealed similar activation in REDD1-proficient and deficient cells (Fig. 4A–C). Moreover, downregulation of FLIP following TG treatment was similar regardless REDD1 expression (Fig. S2A, B). Collectively, these results suggest that the activation of the PERK branch of the UPR was comparable between control and *REDD1/DDIT4* KO cells.

Given that, in response to TG, REDD1-depleted spheroids exhibited a marked increase in TRAILR2/DR5 protein levels compared to REDD1-expressing spheroids (Fig. 3G, H), we wondered whether, despite a similar activation of the PERK pathway, further upregulation of TRAILR2/DR5 could be observed in 2D *REDD1/DDIT4* KO cells in response to ER stress. We assessed TRAILR2/DR5 protein levels in both control and *REDD1/DDIT4* KO cells treated with TG. In contrast to the results for ATF4 and CHOP expression, we observed a significant increase in both TRAILR2/DR5 monomer and oligomer levels in REDD1/DDIT4-deficient cells compared to control cells upon ER stress induction (Fig. 4D, E). TRAILR2/DR5 upregulation was also confirmed in HT29 cells following REDD1 knockdown (Fig. S2C, D). To investigate the mechanism behind the elevated TRAILR2/DR5 expression in *REDD1/DDIT4* KO cells under ER stress, we first measured *TRAILR2/DR5* mRNA levels in both control and *REDD1/DDIT4* KO cells treated with TG. Compared to REDD1/DDIT4-expressing cells, *REDD1/DDIT4* KO cells exhibited a significant upregulation of *TRAILR2/DR5* mRNA upon TG treatment (Fig. 4F, G). Notably, even without ER stress, *REDD1/DDIT4* KO cells showed enhanced *TRAILR2/DR5* mRNA levels (Fig. 4F, G), suggesting that REDD1/DDIT4 plays a role in regulating TRAILR2/DR5 expression in tumor cells, independently of stress conditions.

Although REDD1/DDIT4 expression did not affect CHOP protein levels in response to TG treatment, its essential role in TRAILR2/DR5 induction following ER stress prompted us to investigate whether CHOP was critical for sensitization after REDD1/DDIT4 depletion. CHOP was knocked down using RNA interference (Fig. 4H), and apoptosis was completely inhibited in both control and REDD1/DDIT4-depleted cells (Fig. 4I). Interestingly, analysis of TRAILR2/DR5 expression revealed that CHOP knockdown led to a significant decrease in TRAILR2/DR5 expression (Fig. 4J) in response to TG treatment. However, although not statistically significant, *TRAILR2/DR5* mRNA levels remain higher, in *REDD1/DDIT4* KO cells, regardless of CHOP expression (Fig. 4J, dashed square). This suggests that a CHOP-independent mechanism may be differentially active between control and *REDD1/DDIT4* KO cells, with the latter maintaining higher levels of TRAILR2/DR5 compared to control cells.

During the adaptive phase of the UPR, the IRE1 branch mediates the regulated IRE1α-dependent decay (RIDD) of *TRAILR2/DR5* mRNA through its RNase activity, thereby promoting cell survival [8, 53]. Under unresolved ER stress, IRE1 signaling is attenuated, while PERK signaling persists and ultimately drives apoptotic cell death [54]. We next investigated whether the increased *TRAILR2/DR5* mRNA levels observed in *REDD1/DDIT4* KO cells could result from impaired IRE1 signaling, which may allow for early upregulation of *TRAILR2/DR5* mRNA. To test this, we analyzed IRE1-mediated XBP1 splicing in both control and *REDD1/DDIT4* KO cells following TG treatment. As shown in Fig. S2E, no differences in XBP1 splicing were detected between control and REDD1/DDIT4-deficient cells, suggesting that IRE1 signaling is comparable regardless of REDD1/DDIT4 expression. Although both XBP1 splicing and RIDD are mediated by the IRE1 RNase domain, they are distinct processes [55]. To rule out the involvement of the IRE1 pathway in the upregulation of *TRAILR2/DR5* mRNA in the absence of REDD1/DDIT4, we performed IRE1 knockdown prior to TG treatment and evaluated TRAILR2/DR5 upregulation in both control and *REDD1/DDIT4* KO cells. IRE1 knockdown, confirmed by western blotting and XBP1 splicing analysis (Fig. S2F), slightly facilitated TRAILR2/DR5 upregulation (Fig. S2G). However, this effect was independent of REDD1/DDIT4 expression. These findings suggest that differential IRE1 signaling is unlikely to explain the increased TRAILR2/DR5 expression observed in REDD1/DDIT4-deficient cells.

ER stress also activates c-Jun N-terminal kinase 1 (JNK), which is implicated in apoptosis through the intrinsic pathway [56, 57]. Additionally, REDD1/DDIT4 has been shown to inhibit apoptosis by suppressing JNK signaling in MEFs and retinal precursor cells [58]. Based on these findings, we hypothesized that enhanced JNK signaling might contribute to the increased apoptosis observed in REDD1/DDIT4-deficient cells by promoting TRAILR2/DR5 expression. We confirmed that the loss of REDD1/DDIT4 leads to JNK activation, as evidenced by increased phosphorylation of its target c-Jun at Ser73 (Fig.e S2H, I). However, pharmacological inhibition of JNK did not restore *TRAILR2/DR5* mRNA levels (Fig. S2J), suggesting that JNK signaling does not directly mediate the upregulation of TRAILR2/DR5 in REDD1/DDIT4-deficient cells.

### Identification of the EVI-1/MECOM transcription factor as a potential regulator of TRAILR2/DR5 and apoptosis in REDD1/DDIT4-proficient cells

**Fig. 5:**
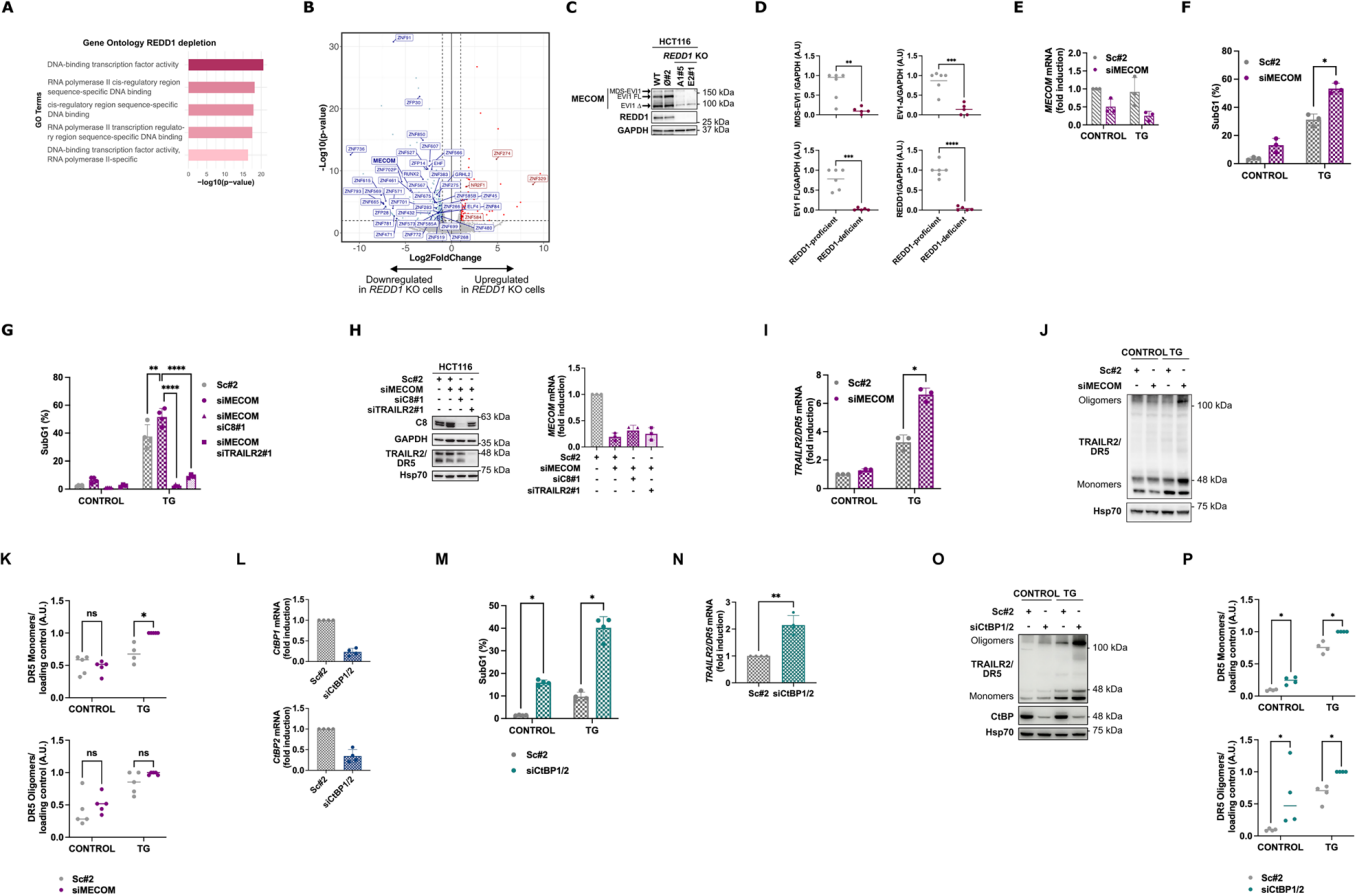
EVI-1/MECOM restrains TRAILR2/DR5 expression in REDD1/DDIT4-proficient cells. **A** Gene Ontology (GO) analysis of significantly dysregulated targets in *REDD1/DDIT4* KO cells identified by RNA-seq. **B** Volcano plot of differentially expressed genes in *REDD1/DDIT4* KO clones versus the REDD1-expressing clone Ø#2. Genes with >|1 | log2fold change and * *P*-value < 0.01 are shown in blue (downregulated) and red (upregulated). Targets associated with the GO term “DNA-binding transcription factor activity” are indicated, with EVI-1/MECOM downregulation highlighted in bold. **C** Validation of EVI-1/MECOM downregulation in *REDD1/DDIT4* KO clones by western blotting in whole cell extracts. GAPDH was used as protein loading control. Representative blot from three independent experiments. **D** Western blot quantification from (**C**): MECOM isoforms and REDD1 levels were normalized to the loading control (*n* = 3. WT and Ø#2 (REDD1-proficient) data were pooled for analysis; *REDD1/DDIT4* KO A1#5 and E2#1 (REDD1-deficient) data were pooled for analysis. **p ≤ 0.01; ***p ≤ 0.001; ****p ≤ 0.0001; Unpaired t test). **E**,**F** HCT116 WT cells were transfected with a scrambled oligonucleotide#2 (Sc#2) or EVI-1/MECOM siRNA (siMECOM) for 48 h before TG (100 nM) treatment. **E** EVI-1/MECOM knockdown was assessed by RT-qPCR. **F** Apoptosis was determined after 24 h treatment by subG1 analysis (*n* = 3 *p ≤ 0.05.; Multiple unpaired t test). **G**,**H** HCT116 WT cells were transfected with scrambled oligonucleotide#2 (Sc#2), EVI-1/MECOM (siMECOM), Caspase-8 (siC8#1) and/or TRAILR2/DR5#1 (siTRAILR2/DR5#1) siRNA, as indicated in the figure, for 48 h before TG (100 nM) treatment for 24 h. **G** Apoptosis was determined after treatment by subG1 analysis (*n* = 4. ****p ≤ 0.0001; **p ≤ 0.01.; Two-way ANOVA. Tukey’s multiple comparisons test). **H** Caspase-8 (C8) and TRAILR2/DR5 knockdowns were confirmed in whole-cell extracts by western blotting. GAPDH and Hsp70 were used as protein-loading controls. EVI-1/MECOM knockdown was assessed by RT-qPCR. **I–L** HCT116 WT cells were transfected with scrambled oligonucleotide#2 (Sc#2) or EVI-1/MECOM siRNA (siMECOM) for 48 h prior to treatment with TG (100 nM) for a further 7 h period. **I** TRAILR2/DR5 expression was assessed by RT-qPCR (*n* = 3. ns not statistically significant; **p ≤ 0.01; Multiple unpaired t test). **J** TRAILR2/DR5 protein levels were determined in whole cell extracts by western blotting. Hsp70 was used as protein-loading control. **K** Western blot quantification from (**J**): TRAILR2/DR5 monomers and oligomers were normalized to the loading control (*n* = 5. ns not statistically significant; *p ≤ 0.05; Multiple unpaired t test). **L–P** HCT116 WT cells were transfected with scrambled oligonucleotide#2 (Sc#2) or CtBP siRNA (siCtBP) for 48 h before TG (100 nM) treatment. **L** CtBP knockdown was assessed by RT-qPCR. **M** Apoptosis was determined after 24 h treatment by subG1 analysis (*n* = 4. *** = *p* ≤ 0.001; Multiple upaired t test) (**N**) TRAILR2/DR5 expression was assessed by RT-qPCR (*n* = 3. ns not statistically significant; **p ≤ 0.01; Multiple unpaired t test). **O** TRAILR2/DR5 protein levels and CtBP knockdown were determined in whole cell extracts by western blotting. Hsp70 was used as a protein-loading control. **P** Western blot quantification from (**O**): TRAILR2/DR5 monomers and oligomers were normalized to the loading control (*n* = 4. *p ≤ 0.05; Multiple unpaired t test).

We hypothesized that the increased sensitivity to ER stress-induced apoptosis observed in the absence of REDD1/DDIT4 is due to elevated expression of TRAILR2/DR5 in these cells, which is markedly higher even under basal conditions (Fig. 4F, G). To find potential mediators of TRAILR2/DR5 expression in REDD1/DDIT4-deficient cells, we treated both REDD1/DDIT4-expressing and *REDD1/DDIT4* KO cells with or without TG for 7 h and performed RNA-seq analysis. Gene ontology analysis of the >200 dysregulated genes common to both *REDD1* KO clones (Fig. S3A) revealed an enrichment of genes involved in transcriptional regulation (Fig. 5A). Among all these hits, we identified the EVI-1/MECOM transcription factor (Fig. 5B) [59] as a potential regulator of TRAILR2/DR5 expression [36]. REDD1/DDIT4-depleted cells exhibited a significant downregulation of EVI-1/MECOM transcript (Fig. 5B). EVI-1/MECOM downregulation was further confirmed by western blot (Fig. 5C). Notably, the different EVI-1/MECOM isoforms (MDS1-EVI1, EVI1-FL, and EVI1-Δ324) were significantly downregulated (Fig. 5D).

To investigate whether the loss of EVI-1/MECOM enhances susceptibility to ER stress-induced apoptosis, we performed EVI-1/MECOM knockdown in HCT116 and HT29 parental cells prior to TG treatment. Knockdown of EVI-1/MECOM (Fig. 5E, S3B) notably increased the sensitivity of cells to TG-induced apoptosis compared to the scrambled RNA controls (Fig. 5F, S3C). Furthermore, similar to the sensitization observed with REDD1/DDIT4 loss, silencing EVI-1/MECOM expression enhanced TG-induced apoptosis in a caspase-8- and TRAILR2/DR5-dependent manner, as this effect was abolished by knockdown of caspase-8 or TRAILR2/DR5 (Fig. 5G, H). A hallmark of the increased susceptibility to ER stress in *REDD1/DDIT4* KO cells was the elevated expression of TRAILR2/DR5 observed following treatment with the ER stress inducer. We then examined whether this effect was also observed in EVI-1/MECOM knockdown cells. As seen in Fig. 5I–K and S3D–F, silencing EVI-1/MECOM expression in REDD1/DDIT4-expressing cells was sufficient to promote TG-induced upregulation of both mRNA (Fig. 5I, S3D) and protein levels of TRAILR2/DR5 (Fig. 5J, K, S3E, F). Collectively, our findings suggest that the loss of EVI-1/MECOM observed in *REDD1/DDIT4* KO cells plays a critical role in regulating TRAILR2/DR5 expression and mediating apoptosis in response to ER stress.

The C-terminal binding protein (CtBP) family of transcriptional corepressors has been shown to cooperate with EVI-1/MECOM in transcriptional repression [31, 32, 60, 61] and is highly expressed in aggressive tumors [36]. Although a direct link between EVI-1/MECOM and TRAILR2/DR5 regulation remains unestablished, CtBP proteins have been implicated as repressors of pro-apoptotic TRAIL receptor expression in tumor cells [36]. Consequently, we investigated the potential role of CtBP1/2 in modulating TRAILR2/DR5 expression and the apoptotic response to ER stress in HCT116 cells. As shown in Fig. 5L–P, silencing CtBP1/2 with siRNA (Fig. 5L) significantly sensitized tumor cells to TG-induced apoptosis (Fig. 5M). This increased sensitivity was associated with a pronounced upregulation of TRAILR2/DR5 at both the mRNA (Fig. 5N) and protein levels (Fig. 5O, P). However, CtBP1/2 role in HT29 cells could not be confirmed (data no shown), which suggest that its contribution is likely cell-type dependent. Overall, our data support the hypothesis that EVI-1/MECOM may function as a key repressor of the apoptotic response to chronic ER stress in colorectal tumor cells, by counteracting the upregulation of TRAILR2/DR5, mediated by the PERK-ATF4-CHOP signaling axis of the UPR. The loss of EVI-1/MECOM expression in *REDD1/DDIT4* KO cells results in impaired repression of TRAILR2/DR5 gene expression. This regulation, which may occur through CtBP family proteins, leads to an elevation of TRAILR2/DR5 levels in these tumor cells, and facilitates the execution of cell death, via caspase-8-dependent apoptosis, when tumor cells face ER stress.

## Discussion

In a tumor, cancer cells face several insults, including nutrient starvation, hypoxia or ROS. Consequently, the function of protein folding machinery is impaired in cancer cells leading to the accumulation of unfolded and misfolded proteins and thus to ER stress [1]. Chronic or excessive ER stress switches UPR signaling from an adaptive response to trigger pro-apoptotic mechanisms [5, 6, 45]. In different cellular models, including HCT116 colorectal cancer cells, unmitigated ER stress activates caspase-8-dependent apoptosis through the axis PERK-P-eIF2α-ATF4-CHOP-TRAILR2/DR5 [8, 9, 13].

**Fig. 6:**
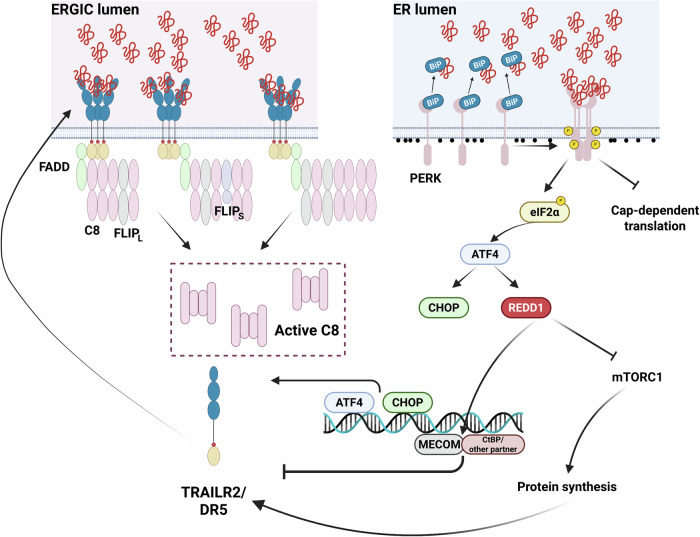
Schematic representation of the proposed mechanism through which REDD1/DDIT4 regulates ER stress-induced upregulation of TRAILR2/DR5 expression and apoptosis in colorectal tumor cells. The figure illustrates the critical role of REDD1/DDIT4 in regulating TRAILR2/DR5-induced caspase-8 activation and apoptosis under chronic ER stress, by inhibiting mTORC1 activity and promoting EVI-1/MECOM-mediated repression of TRAILR2/DR5 gene expression. Created with BioRender.

Although REDD1/DDIT4 was initially described as a hypoxia-induced protein [24], other stress situations, including ER stress, result in REDD1/DDIT4 up-regulation [62, 63]. REDD1/DDIT4 function in tumourigenesis is controversial since tumor suppressor [24–26] and pro-tumoural [27, 37, 64] roles have been associated with REDD1/DDIT4 induction. In this respect, elevated levels of REDD1/DDIT4 were significantly associated with a worse prognosis in several malignancies including colon cancer [65, 66]. Our results in colon cancer cell lines point to an adaptive function of REDD1/DDIT4 when they face chronic and excessive ER stress by repressing pro-apoptotic TRAILR2/DR5 receptor expression. REDD1/DDIT4 function has been mainly linked to the control of mTORC1 activity under stress through the TSC1/2 complex [15, 24, 27, 42]. Our data point to a role of sustained mTORC1 activation in the increased sensitivity of REDD1-deficient tumor cells to ER stress. Constitutive mTORC1 activation by the loss of TSC1/2 signaling has been reported to induce apoptosis by stimulating the IRE1-ASK1-JNK pathway [67]. Although, we cannot completely rule out the involvement of this signaling pathway in the sensitization of REDD1-deficient cells to apoptosis following ER stress [27], our findings in IRE1 knockdown cells and with the JNK inhibitor SP600125 likely exclude the involvement of this pathway in the control of TRAILR2/DR5 levels by REDD1/DDIT4. Activation of the PERK branch of the UPR and the resulting FLIP down-regulation and TRAILR2/DR5 upregulation are key events for apoptosis induction in colorectal tumor cells facing ER stress [8, 11]. However, our data indicate that REDD1-proficient and deficient cells exhibit similar PERK pathway dynamics, as indicated by the expression level of ATF4 and CHOP transcription factors. Likewise, in cells undergoing sustained ER stress, FLIP loss was independent of REDD1/DDIT4 expression, suggesting that the enhanced caspase-8 activation and apoptosis observed in REDD1/DDIT4 deficient cells are probably not linked to faster downregulation of FLIP levels upon ER stress. We demonstrate that REDD1/DDIT4 deficiency led to a greater increase in TRAILR2/DR5 expression through a CHOP-independent mechanism. Overall, our findings suggest that REDD1/DDIT4 functions as a brake on the early upregulation of TRAILR2/DR5, thereby delaying caspase-8 activation and the induction of the apoptotic program in response to chronic ER stress (Fig. 6). In this regard, our results represent the first identification of the transcriptional regulator EVI-1/MECOM as a key modulator of TRAILR2/DR5 expression. In the HCT116 cellular model, EVI-1/MECOM acts with its well-known partner CtBP [31, 32, 60, 61]. Notably, both EVI/MECOM and CtBP are often overexpressed in human colon cancer cells [68, 69]. However, CtBP contribution, could not be confirmed in the HT29 model, indicating that its role may be cell-type dependent. Notably, EVI-1/MECOM interactome involves other proteins linked to transcriptional repression. Some examples include histone deacetylase (HDAC) 1 or HDAC2 [60, 70], the histone methyltransferase SUV39H1 [71] or MBD3b, a member of the Mi-2/NuRD transcriptional repressor complex [72]. Thus, cell type-dependent specificities may determine which factor(s) operate with EVI-1/MECOM to mediate TRAILR2/DR5 transcriptional repression.

Further studies aimed at elucidating the molecular mechanism behind the regulation of EVI-1/MECOM expression in colon tumor cells by REDD1/DDIT4 will be crucial to gaining a broader understanding of the various mechanisms involved in tumor cell responses to ER stress. A deeper comprehension of how REDD1/DDIT4, in conjunction with EVI-1/MECOM, contributes to the escape of tumor cells from apoptosis activation is a critical question that could uncover new therapeutic targets. Additionally, our data reinforce the role of REDD1/DDIT4 in cellular adaptation when colorectal cancer cells, organized in a 3D spatial configuration, face stress, suggesting that REDD1/DDIT4 may serve as a promising target to enhance the efficacy of therapies that aim to boost pro-apoptotic UPR signaling during ER stress.

## Materials and Methods

### Cell culture and reagents

Human colorectal carcinoma cell line HCT116 (American Type Culture Collection) was kindly donated by Dr. J.A. Pintor-Toro (Andalusian Center for Molecular Biology and Regenerative Medicine-CABIMER, Seville, Spain). HT-29 colon cancer cell line was obtained from Cell Lines Service GmbH (CLS, Germany). HCT116 and HT29 cell cultures were maintained in McCoy’s 5 A modified medium with 2 mM L-glutamine, penicillin (50 U/ml), streptomycin (50 μg/ml) and 10% fetal bovine serum. HEK293T cells, a donation of Dr. A. Rodriguez (Autonomous University of Madrid, Spain), were maintained in DMEM medium supplemented with 10% fetal bovine serum (Gibco), 2mM L-glutamine, 50 U of penicillin/ml and 50 μg of streptomycin/ml. Cells were grown at 37 °C in a 5% CO_2_-humidified, 95% air incubator and regularly tested for mycoplasma contamination.

The ER stress-inducers thapsigargin and tunicamycin, JNK pharmacological inhibitor SP600125, ethidium bromide and Ribonuclease A (RNase A) were purchased from Sigma. Q-VD-Oph (QVD) was obtained from Apexbio and Torin-1 was purchased from TOCRIS Bioscience.

### CRISPR/Cas9 editing

*REDD1/DDIT4* KO HCT116 cells were generated by CRISPR/Cas9-based gene targeting. Two different guide RNAs (gRNAs) were designed: one targeting exon 1, near the ATG initiator codon region, and the other targeting exon 2. Briefly, forward and reverse oligos for the gRNA against REDD1/DDTI4 were annealed and ligated into pSpCas9(BB)-2A-GFP (PX458) (#48138, Addgene). 48 h post-transfection, GFP-positive cells were sorted using a BD FACSAria cell sorter (BD5 FACSAriaTM III, BD Biosciences, Heidelberg, Germany). Finally, REDD1 depletion of cultured clones was confirmed by western blotting.

**Table:** 

Name	Sequence (5’ → 3’)
REDD1-ATG-gRNA1 Fwd	CACCGCGACGAGAAGCGGTCCCAA
REDD1-ATG-gRNA1 Rev	AAACTTGGGACCGCTTCTCGTCGC
REDD1-Ex2-gRNA2 Fwd	CACCGAGGCATCAGCAGGCGCGCA
REDD1- Ex2-gRNA2 Rev	AAACTGCGCGCCTGCTGATGCCTC

### Generation of HCT116 REDD1 KO-derived cell lines

pBABE-puro-ø plasmid was kindly provided by Dr. Cristina Muñoz-Pinedo (IDIBELL, Barcelona), pBABE-FLIP_L_ plasmid was produced in our lab [73] and pBABE-HA-REDD1 was purchased from Addgene (#133550). For stable knockdown experiments, shRNAs against caspase-8 or TRAILR2/DR5 in a pSUPER vector (OligoEngine) were digested and cloned between *EcoR1 and Cla1* into an H1 promoter-driven GFP-encoding pLVTHM lentiviral vector [74]. Viral production was achieved by transfection of HEK293T cells using the calcium phosphate method. DNA was transfected in proportion 1:2:3 containing pCI-VSV-G:pVpack-GP-dl:transfer vector or pMD2.G:psPAX2:transfer vector, according to retroviral and lentiviral production, respectively. Retro- or lentiviruses-containing supernatants were collected 48 h after transfection and concentrated by ultracentrifugation at 22,000 rpm for 90 min at 4 °C. Tumor cells were plated at 6 × 10^5^ cells per 10-cm dish and infected with the viruses mentioned above 2 days later. Stable populations of tumor cells infected with retroviruses were obtained after selection in a culture medium containing puromycin (1.5 µg/ml) for at least 48 h. Lentiviral infection efficiency was assessed by GFP expression, which was examined by flow cytometry using a BD FACScalibur flow cytometer

**Table:** 

shRNA	Sequence (3’→5’)
**shCaspase8#1 (shC8#1)**	5’GATCCCC**GGAGCTGCTCTTCCGAATT**TTCAAGAGA**AATTCGGAAGAGCAGCTCC**TTTTTA3’
**shScrambled (shScr)**	5′GATCCCC**CTTTGGGTGATCTACGTTA**TTCAAGAGA**TAACGTAGATCACCCAAAG**TTTTTA3’
**shTRAIL-R2#1**	5′GATCCCC**GACCCTTGTGCTCGTTGTC**TTCAAGAGA**GACAACGAGCACAAGGGTCT**TTTTTA3′

### Multicellular tumor spheroids (MCTs)

MCTs were generated as described before [52]. For generation of HCT116-derived spheroids, cells were seeded into Terasaki multiwell plates (100 cells/well) (Greiner Bio-One, Frickenhausen, Germany) and placed in humid chambers in the incubator. After 3 days of cultivation, spheroids were transferred to agarose-coated 96-well plates (F-bottom, Greiner Bio-One, Frickenhausen, Germany). Medium was changed every second to third day until spheroids reached a diameter of approximately 500 μm. Then, spheroids were treated as indicated in figure legends.

### Monitoring of MCTSs growth

To estimate the growth of the generated spheroids, transmitted light photos were taken daily with a Leica inverted digital microscope (Leica DFC500). Then, the areas of the spheroids were determined using the ImageJ software. It was assumed that the generated MCTSs are spheres and, therefore, their diameters could be calculated according to the following equation:

$$d=2.\sqrt{\frac{A}{\pi }}$$

Where A is the measured area of the spheroid and d is the diameter.

### Treatment of MCTSs

The day of treatment spheroids were transferred to a new F-bottom 96-well plate coated with agarose in a volume of 100 μL, using a yellow cut tip. Afterwards, 100 μL/well of fresh medium containing the appropriate drug (2X concentrated) were added.

### Analysis of hypodiploid apoptotic cells (SubG1 population)

Cells (1.5 × 10^5^/well) were seeded into 6-well plates and two days later treated as indicated in the figure legends. After treatment, hypodiploid apoptotic cells were detected by flow cytometry according to published procedures [75]. Briefly, cells were detached and dissociated with trypsin/EDTA and washed with cold PBS, fixed in 70% cold ethanol, and then stained with propidium iodide (40 μg/mL) while treating with RNase (100 μg/mL) for 30 min in the dark. Quantitative analysis of the subG1 population was carried out in a FACSCalibur cytometer using the Cell Quest software (Becton Dickinson, Mountain View, CA, USA) or LSRFortessa X-20 cytometer using the BD FACSDiva™ Software (Becton Dickinson, Mountain View, CA, USA).

### Analysis of apoptosis by Annexin V-FITC/PI staining

MTCs were washed with temperate PBS and dissociated using trypsin/EDTA. Single-cell suspensions were stained with Annexin V-FITC (Immunostep, Salamanca, Spain) and propidium iodide (20 μg/mL, Sigma-Aldrich, MO, USA) in Annexin V binding buffer (10 mM HEPES/NaOH (pH 7.4), 140 mM NaCl, 2.5 mM CaCl_2_) for 15 min at room temperature in the dark. 400 µL of additional Annexin V binding buffer was added to each tube before analysis using a FACSCalibur cytometer (Becton Dickinson, Mountain View, CA, USA). Quantification of apoptotic cells was accomplished using Cell Quest software (Becton Dickinson, Mountain View, CA, USA).

### Immunoblot analysis of proteins

Cells were washed with phosphate-buffered saline (PBS) and subsequently lysed in TR3 buffer (10 mM Na_2_HPO_4_, 10% glycerol, 3% SDS). Protein concentration was determined using the DC (detergent-compatible) protein assay reagent (Bio-Rad Laboratories, USA), following which loading buffer was added. Proteins were then separated by SDS-polyacrylamide gel electrophoresis (SDS-PAGE) using mini-gels, and detection was carried out as previously described [73]. GAPDH, Hsp70, and α-tubulin were used as loading controls for protein normalization. Quantifications were performed with the Image LabTM 6.0 software (Bio-Rad Laboratories, Inc., CA, USA) or ImageJ software [76].

**Table:** 

Antigen	Dilution	Provider	Reference
**4EBP1**	1:1000	Cell Signaling Tech	9452
**AKT**	1:1000	Cell Signaling Tech	9272
**ATF4**	1:1000	Santa Cruz Biotech	SC-200
**ATF4**	1:1000	Cell Signaling Tech	11815
**Caspase-8**	1:1000	Cell Signaling Tech	9746
**CtBP**	1:2000	Santa Cruz Biotech	sc-17759
**CHOP**	1:1000	Cell Signaling Tech	5554
**FLIP (7F10)**	1:1000	ENZO	ALX-804-961
**GAPDH**	1:40000	Santa Cruz Biotech	SC-47724
**HRP-linked antibody**	1:5000	DAKO	P0448
**HRP-linked antibody**	1:5000	DAKO	P0447
**HRP-linked antibody**	1:5000	DAKO	P0449
**Hsp70**	1:20000	Merck	H5147
**IRE1α**	1:1000	Cell Signaling Tech	3294
**MECOM**	1:1000	Cell Signaling Tech	2593
**p70 S6K**	1:1000	Cell Signaling Tech	9202S
**Phospho-4EBP1 (S65)**	1:1000	Cell Signaling Tech	9451
**Phospho-c-Jun (S73)**	1:1000	Cell Signaling Tech	3270
**Phospho-p70 S6K (T389)**	1:1000	Cell Signaling Tech	9206S
**REDD1**	1:2000	Proteintech	10638-1-AP
**TRAILR2/DR5**	1:2000	R&D Systems	AF631
**α-tubulin**	1:40000	Santa Cruz Biotech	SC-23948

### RNA interference

For HCT116 cells, siRNA-mediated knockdown was carried out with jetPRIME® transfection reagent (POLYplus transfection) using 50 nM of siRNA (Sigma) according to the manufacturer´s instructions. Generally, 1,5 ×10^5^ cells/well were seeded into 6-well plates and transfected while in suspension. The following day, the medium was carefully replaced with fresh medium and cells were incubated for 24 h. Then, cells were treated as indicated in the figure legends. HT29 cells were transfected with 50 nM of siRNA using DharmaFECT-1 (Dharmacon) as described by the manufacturer. Generally, 1,5 ×10^5^ cells/well were seeded into 6-well plates and transfected the day after. After 6 h, transfection medium was replaced with fresh medium, and cells were incubated until next day. Then, cells were treated as indicate in figure legends. Knockdowns were confirmed by western blot or RT-qPCR, as indicated.

**Table:** 

siRNA	Sequence 5’ → 3’
**ATF4**	GCCUAGGUCUCUUAGAUGA[dT][dT]
**Caspase-8#1**	GGAGCUGCUCUUCCGAUU [dT][dT]
**CHOP**_**POOL**_	AGGGAGAACCAGGAAACGGAA[dT][dT] ACGGCTCAAGCAGGAAATCGA[dT][dT] AAGGAAGTGTATCTTCATACA[dT][dT] CAGCTTGTATATAGAGATTGT[dT][dT]
**CTBP**	GGGAGGACCUGGAGAAGUU [dT] [dT]
**IRE1α**	GCGUCUUUUACUACGUAAU [dT] [dT]
**MECOM**	GAAUGAACACUCCAUAGAAAC[dT] [dT]
**REDD1#1**	GUGGAGACUAGAGGCAGGAGC[dT][dT]
**REDD1#2**	GAUACUCACUGUUCAUGAA [dT][dT]
**REDD1#3**	ACGCAUGAAUGUAAGAGUA[dT][dT]
**REDD1**_**POOL**_	GUGGAGACUAGAGGCAGGAGC[dT][dT] GAUACUCACUGUUCAUGAA [dT][dT] ACGCAUGAAUGUAAGAGUA[dT][dT]
**Scrambled (Sc)**	UGGUUUACAUGUCGACUAA[dT] [dT]
**Scrambled#2 (Sc#2)**	CUUUGGGUGAUCUACGUUA[dT][dT]
**Scambled**_**POOL**_ **(Sc**_**PooL)**_	UGGUUUACAUGUCGACUAA[dT][dT] UGGUUUACAUGUUGUGUGA[dT][dT] UGGUUUACAUGUUUUCUGA[dT][dT] UGGUUUACAUGUUUUCCUA[dT][dT]
**TRAILR1/siDR4#1**	GGAACUUUCCGGAAUGACA[dT][dT]
**TRAILR2/siDR5#1**	GACCCUUGUGCUCGUUGUC[dT][dT]

### RNA extraction

RNA extraction was carried out with PRImeZOL reagent (Canvax Biotech), following the manufacturer’s instructions. The RNA pellet was resuspended in 20-60 μL of DEPC-treated H_2_O. After 5 min at RT, tubes were heated at 60 °C for 10 min in a heat block. RNA samples were spun down and placed on ice. RNA concentration was determined using a NanoDrop spectrophotometer ND-100 (Thermo Fisher).

### Semiquantitative RT-PCR (sqRT-PCR) for analysis of XBP1 splicing

1 μg of total RNA was retrotranscribed using iScript cDNA Synthesis kit (BioRad, 1708891) according to the manufacturer´s instructions. Next, complementary DNA (cDNA) was amplified by PCR with specific primers obtained from Sigma. PCR products of XPB1 and β-actin fragments were visualized on 3 or 1% agarose gels, respectively, in 1X TAE buffer with 0.5 μg/mL of ethidium bromide.

**Table:** 

Primers	Sequence 5’ → 3’
**XBP1-forward**	TTACGGGAGAAAACTCACGGC
**XBP1-reverse**	GGGTCCAACTTGTCCAGAATGC
**β-actin-forward**	TGACGGGGTCACCCACACTGTGCCCATCTA
**β-actin-reverse**	CATGAAGCATTTGCGGTGGACGATGGAGGG

### Real time-qPCR

Retrotranscription was performed as described in the previous section. RT-qPCR for TRAILR2/DR5 expression was carried out in triplicates for each sample with specific TaqMan probes and 2x FastStart Universal Probe Master (ROX) (Roche, 04913957001) following the manufacturer’s instructions.

**Table:** 

Taqman probe	Reference
**HPRT**	Hs01003267_m1
**TRAILR2/DR5**	Hs00366278_m1

In case of EVI-1/MECOM and CtBP1/2 expression, cDNA and the corresponding primers were mixed with 2x iTaq™ Universal SYBR® Green Supermix (BioRad, 1725121) following the manufacturer’s instructions. Each sample was also run in triplicates.

**Table:** 

Primers	Sequence 5’ → 3’
**GAPDH-forward**	ATGGGGAAGGTGAAGGTCG
**GAPDH-reverse**	GGGTCATTGATGGCAACAATATC
**MECOM-forward**	AGTGCCCTGGAGATGAGTTG
**MECOM-reverse**	TTTGAGGCTATCTGTGAAGTGC
**CtBP1-forward**	GACAGCCTGAAGAACTGTGTC
**CtBP1-reverse**	TATAGGCAGCCCCATTGAGCT
**CtBP2-forward**	TTCAAGGCCCTGAGAGTGAT
**CtBP2-reverse**	GAGTCCGCTGTCTCTTCCAC

DEPC-treated water instead of cDNA was run additionally to detect possible contaminations, and qPCR plates (Nerbeplus, 04-083-0150) were spun down before introducing them into devices. qPCRs were performed in 7500 Real-Time PCR System or QuantStudio^TM^ 5 Real-Time PCR System (Applied Biosystems) according to comparative C_T_ protocol. HPRT or GAPDH were used as internal controls and RNA expression levels were given as a fraction of RNA levels in control cells.

### RNA sequencing

Total RNA was isolated using RNeasy Plus Mini Kit (QIAGEN, Hilden Germany), following the manufacturer’s instruction. The remaining DNA was further depleted by DNase treatment (TURBO DNase, Invitrogen). Libraries were prepared with the Illumina stranded Total RNA prep with Ribo Zero Plus (Illumina, San Diego, CA, USA) and sequencing was performed with a NovaSeq6000 SP system (Illumina) with 50 bp paired-end reads with the Genomic Unit of CABIMER (Seville, Spain). Two biological replicates for each condition were sequenced. The downstream analysis was performed by Novogene (Cambridge, UK). Reads were aligned to human genomes (GRCh38.p12/hg38) using the HISAT2 alignment program [77]. Gene expression levels for each condition were estimated using the Fragments Per Kilobase of transcript sequence per Million base pairs sequenced (FPKM) method [78]. Finally, samples were normalized using the DESeq method, and gene expression differential analysis was conducted with the DESeq2 software [79]. Differentially expressed genes with *p*-values < 0.01 and Log2FC > 1 (upregulated genes) or Log2FC < − 1 (downregulated genes) were selected for further analysis.

### Statistical analysis

Statistical analysis was performed using GraphPad Prism 8 software (GraphPad Software Inc.). Quantitative data are presented as mean values ± standard deviation (SD) from at least three independent experiments. In cases where only two experiments were conducted, this is indicated in the figure legends, and data are presented as mean values ± standard error of the mean (SEM). Statistical significance between groups was assessed using the appropriate test specified in the figure legends. Significance levels are indicated by asterisks: ns = not statistically significant; * = *p* ≤ 0.05; ** = *p* ≤ 0.01; *** = *p* ≤ 0.001; **** = *p* ≤ 0.0001.

## Supplementary information

Supplementary Information

Supplementary Information

Supplementary Information

Supplementary Information

Supplementary Information

## Data Availability

All data generated or analyzed during this study are included in the main text and the supplementary information files. RNA-seq data associated with this research are deposited at the EMBL Nucleotide Sequence Database (ENA) under the accession number PRJEB105045.
