# Supplementary material for: REDD1/DDIT4 counteracts endoplasmic reticulum stress-induced apoptosis by controlling the expression of death receptor TRAILR2/DR5 in cancer cells
Source: Cell Death Dis. 2026 Mar 28;17(1):425. doi: 10.1038/s41419-026-08648-7 (PMC13153209; doi:10.1038/s41419-026-08648-7)
Supplement: Supplementary file 1 — Supplementary Information [file 41419_2026_8648_MOESM1_ESM.docx]

**Supplementary figure legends**

**Figure S1. Validation of increasing induction of apoptosis upon ER stress in REDD1 knockdown cells.** **(A-C)** HCT116 cells were transfected with scrambled oligonucleotide (Sc) or REDD1 siRNAs siREDD1#1, siREDD1#2 or siREDD1#3 for 48 h prior to treatment. Apoptosis was determined after treatment by subG1 analysis. **(A)** HCT116 Sc, siREDD1#1 and siREDD1#3 cells were treated with TG (100 nM) for 24 h (n = 3. ns = not statistically significant; *p ≤ 0.05; ***p ≤ 0.001. Two-way ANOVA. Tukey’s multiple comparisons test). **(B-C)** HCT116 Sc and siREDD1#2 **(B)** or siREDD1#3 **(C)** cells were treated with TN (1 μg/mL) for 30 h (n = 3. ns = not statistically significant; *p ≤ 0.05; Multiple unpaired t test). **(D)** REDD1 knockdown and 4EBP1 phosphorylation after 7 h of TG treatment were assessed in whole cell extracts by western blot. α-tubulin was used as protein-loading control. **(E-F)** Quantification of western blots from **(D)**: REDD1 **(E)** was normalized to the loading control, and phospho-4EBP1 **(F)** to its total protein levels (n = 4. Data from siREDD1#1 and siREDD1#3 groups were pooled for analysis. *p ≤ 0.05; Multiple unpaired t test). **(G-H)** REDD1 knockdown using siREDD1#2 **(G)** or siREDD1#3 **(H)**. Phosphorylation of 4EBP1 and p70S6K were assessed after 7 h of TN treatment. GAPDH served as a loading control. **(I-K)** Quantification of western blots from **(G-H)**: REDD1 **(I)** was normalized to the loading control, and phospho-4EBP1 **(F)** and phopho-p70S6K **(K)** to their total protein levels (n = 4. Data from siREDD1#2 and siREDD1#3 groups were pooled for analysis. ns = not statistically significant; p ≤ 0.05; Multiple unpaired t test). **(L)** HT29 cells were transfected with scrambled oligonucleotide (Sc) or a pool of REDD1 siRNA for 24 hours, prior treatment with or without TG (1 µM), for 48 hours. Apoptosis was quantified by subG1 analysis (n = 3. ns = not statistically significant; *p ≤ 0.05; Multiple unpaired t test). **(M)** REDD1 knockdown was confirmed by western blotting in whole cell extracts, with GAPDH as a loading control, after 24 h treatment with TG. **(N)** Quantification of western blot from **(M)**: REDD1 was normalized to the loading control. **(O)** HCT116 WT cells and ø#2 clone, as control cells, and *REDD1/DDIT4 KO* E2#1 clone carrying pBABE-ø or pBABE-HA-REDD1 vectors were treated or not with TG (100 nM) for 24 h. Apoptosis was determined after treatment by subG1 analysis (n = 4. ***p ≤ 0.001; ****p ≤ 0.0001; Two-way ANOVA. Tukey’s multiple comparisons test). **(P)** REDD1 levels from **(O)** were assessed in whole cell extracts by western blotting. α–tubulin was used as protein-loading control. **(Q)** HT29 cells were transfected as in **(L)**, and then, treated with or without Torin-1 (250 nM) for 2 h, followed by the addition of TG (1 µM) for a further 24 h-period. REDD1 levels and p70S6K phosphorylation were determined in whole cell extracts by western blotting. GAPDH and α-tubulin were used as protein-loading controls. **(R)** Quantification of western blots from **(Q)**: phospho-p70S6K was normalized to its total protein levels (n = 3. ns = not statistically significant; ****p ≤ 0.0001; Two-way ANOVA with Tukey’s multiple comparisons test). **(S)** HT29 cells were transfected as in **(L)**, and then, treated with or without Torin-1 (250 nM) for 2 h, followed by the addition of TG (1 µM) for a further 48 h-period. Apoptosis was determined subG1 analysis (n = 3. *p ≤ 0.05; **p ≤ 0.01; ***p ≤ 0.001; two-way ANOVA with Tukey’s multiple comparisons test).

**Figure S2. Analysis of FLIP_L_ in HCT116 REDD1-deficient cells, TRAILR2/DR5 upregulation in the HT29 cellular model, and assessment of the role of the IRE1α branch of the UPR on the increased susceptibility of *REDD1/DDIT4 KO* cells to ER stress-induced TRAILR2/DR5 upregulation. (A-B)** HCT116 control clone (ø#2) and *REDD1/DDIT4* KO clones A1#5 and E2#1 were treated with TG (100 nM) for the indicated times. **(A)** FLIP_L_ levels were assessed in whole cell extracts by western blot. GAPDH was used as a loading control. **(B)** FLIP_L_ were normalized to the loading control (n= 4. Data from *REDD1/DDIT4* KO A1#5 and E2#1 groups were pooled for analysis. ns = not statistically significant. Multiple unpaired t test). **(C-D)** HT29 cells were transfected with scrambled oligonucleotide (Sc) or a pool of REDD1 siRNA for 24 hours, followed by treatment with or without TG (1 µM), for 24 hours. **(C)** TRAILR2/DR5 levels were assessed in whole cell extracts by western blotting. **(D)** TRAILR2/DR5 levels were normalized to the loading control (n = 4. ns = not statistically significant; *p ≤ 0.05; Multiple unpaired t test). **(E)** HCT116 ø#2 and *REDD1/DDIT4* KO A1#5 clones were treated with or without TG (100 nM) for the indicated times. XBP1 splicing and β-actin levels were examined by semiquantitative RT-PCR (sqRT-PCR) (C- = negative control). **(F)** HCT116 ø#2 and *REDD1* KO A1#5 clones were transfected with scrambled oligonucleotide#2 (Sc) or IRE1α siRNA (siIRE1α) for 48 h prior to TG (100 nM) treatment for a further 7 h (n = 2). REDD1 deletion and IRE1α knockdown were determined in whole cell extracts by western blotting. Hsp70 was used as protein-loading control. IRE1α knockdown efficiency was also assessed through analysis of XBP1 splicing by RT-PCR. **(G)** *TRAILR2/DR5* expression was examined by RT-qPCR. **(H-J)** HCT116 ø#2, *REDD1* KO A1#5 and E2#1 cells were treated with or without the JNK inhibitor SP600125 (25 μM) for 2 h and then TG (100 nM) was added or not for a further 16 h-period (n = 2). **(H)** Phosphorylation of c-Jun and REDD1 levels were determined in whole-cell extracts by western blotting. α-tubulin was used as protein-loading control. **(I)** Quantification from **(H)**: phospho-c-Jun levels were normalized to the loading control. **(J)** TRAILR2/DR5 expression was assessed by RT-qPCR.

**Figure S3. Identification of EVI-1/MECOM as a regulator of TRAILR2/DR5 expression in colorectal cancer cells. (A)** Venn diagram showing the intersection of dysregulated genes in *REDD1/DDIT4* KO clones versus the REDD1-expressing clone Ø#2, treated without (left panel) or with TG (100 nM) (right panel) for 7h. **(B-F)** HT29 cells were transfected with either a scrambled oligonucleotide (Sc) or a EVI-1/MECOM siRNA (siMECOM) for 24 hours, followed by treatment with or without TG (1 µM). **(B)** EVI-1/MECOM knocked was confirmed by western blotting in whole-cell extracts. GAPDH was used as a protein-loading control. **(C)** Apoptosis was quantified by subG1 analysis after 48 h of TG treatment (n = 3. ns = not statistically significant; *p ≤ 0.05; Multiple unpaired t test). **(D)** TRAILR2/DR5 expression was assessed by RT-qPCR (n = 6. ns = not statistically significant; **p ≤ 0.01; Multiple unpaired t test). **(E)** TRAILR2/DR5 protein levels were determined in whole cell extracts by western blotting. Hsp70 was used as protein-loading control. **(F)** Western blot quantification from **(E):** TRAILR2/DR5 monomers and oligomers were normalized to the loading control (n = 6. ns = not statistically significant; *p ≤ 0.05; Multiple unpaired t test).
