# Supplementary figures and images for: REDD1/DDIT4 counteracts endoplasmic reticulum stress-induced apoptosis by controlling the expression of death receptor TRAILR2/DR5 in cancer cells
Source: Cell Death Dis. 2026 Mar 28;17(1):425. doi: 10.1038/s41419-026-08648-7 (PMC13153209; doi:10.1038/s41419-026-08648-7)

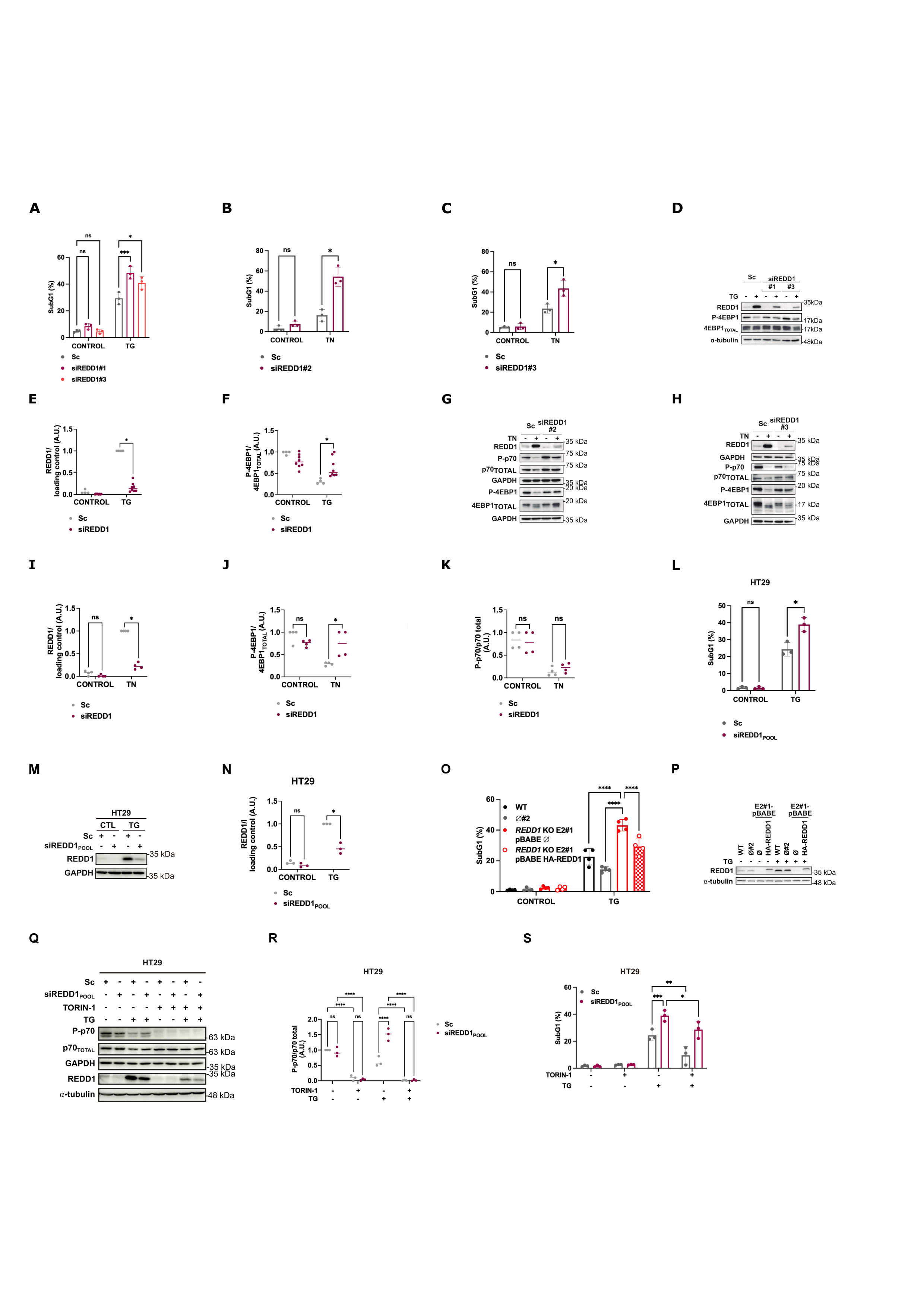

Supplement: Supplementary file 2 — Supplementary Information [file 41419_2026_8648_MOESM2_ESM.tif]

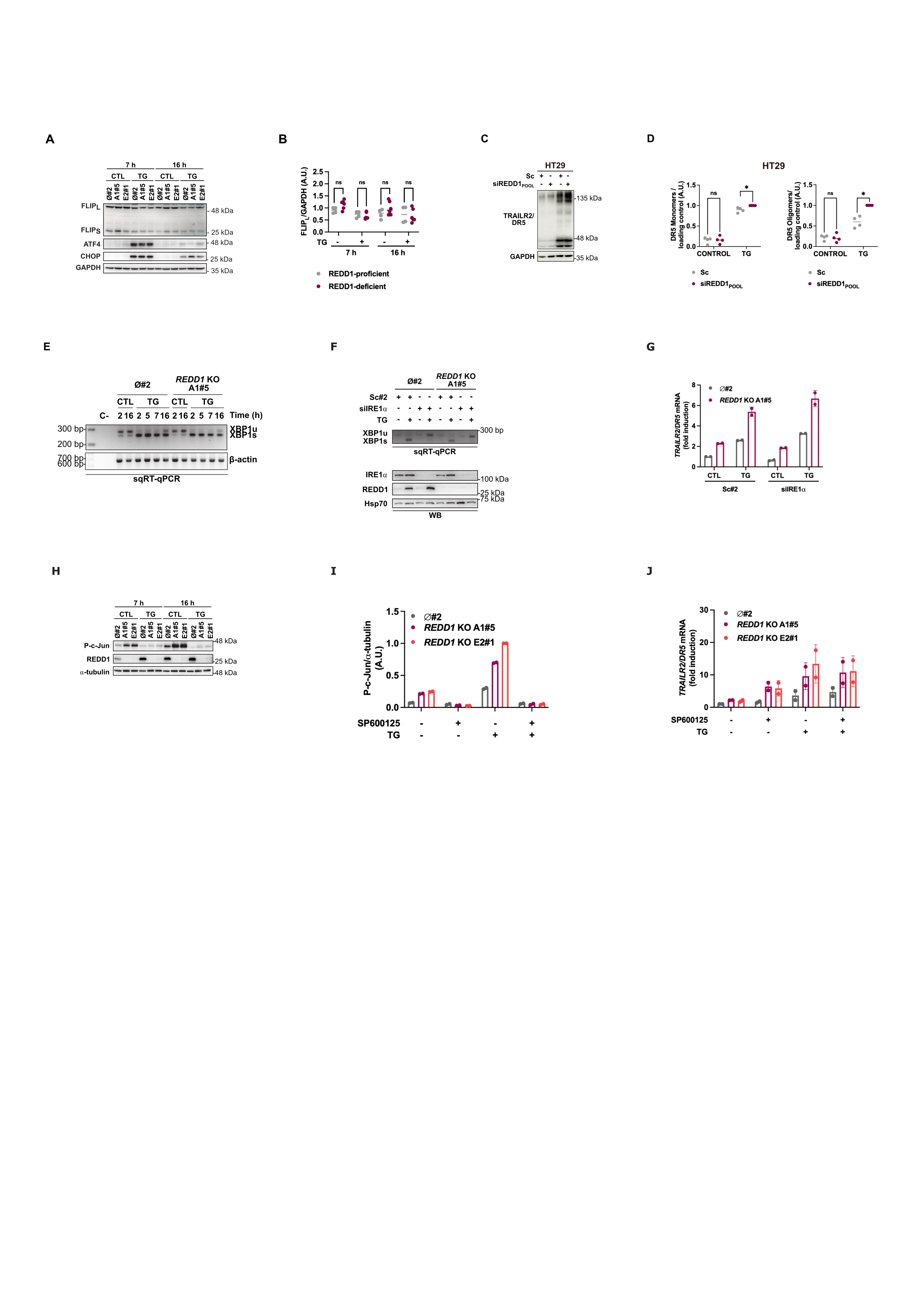

Supplement: Supplementary file 3 — Supplementary Information [file 41419_2026_8648_MOESM3_ESM.tif]

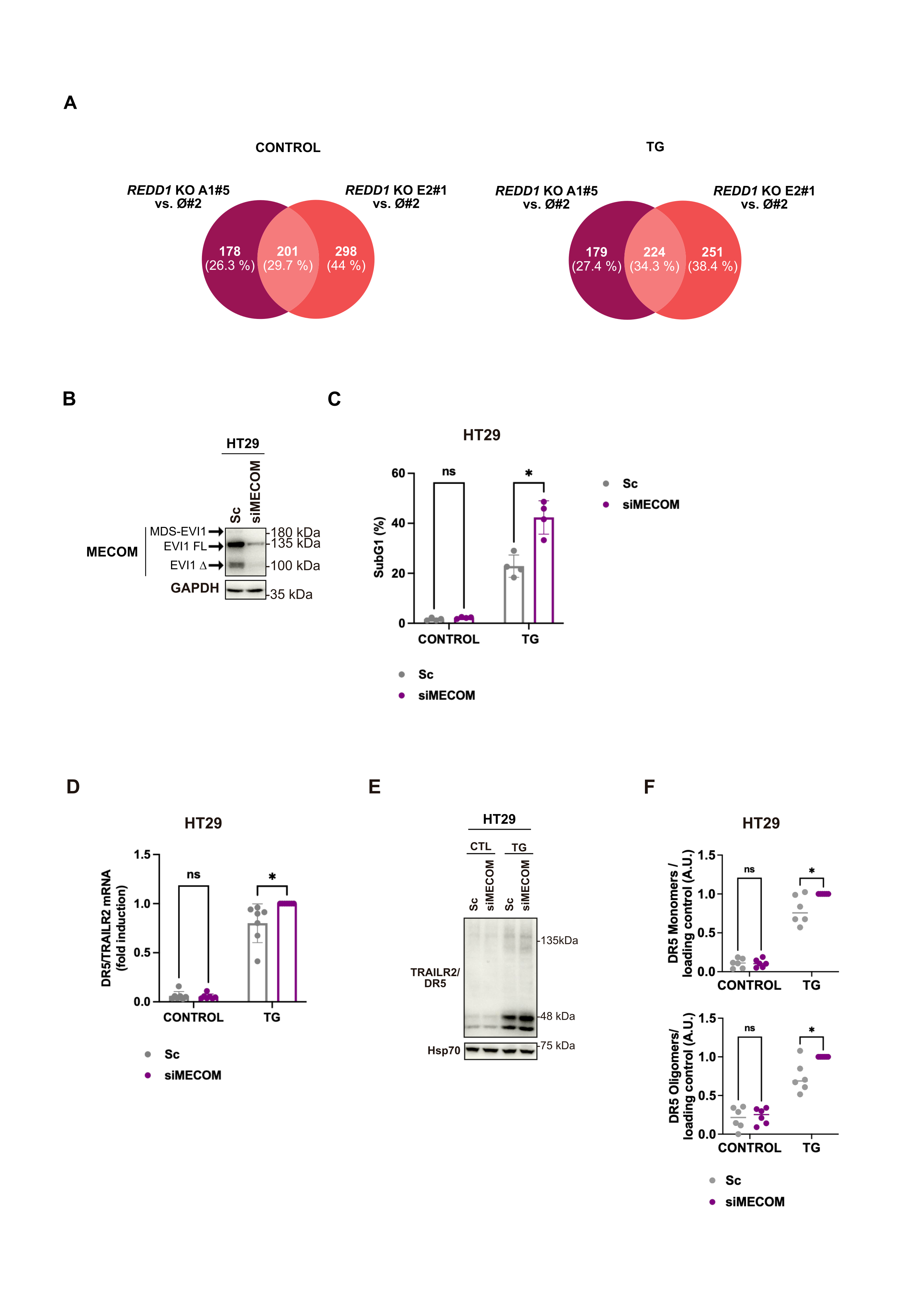

Supplement: Supplementary file 4 — Supplementary Information [file 41419_2026_8648_MOESM4_ESM.tif]
