# Supplementary material for: REDD1/DDIT4 counteracts endoplasmic reticulum stress-induced apoptosis by controlling the expression of death receptor TRAILR2/DR5 in cancer cells
Source: Cell Death Dis. 2026 Mar 28;17(1):425. doi: 10.1038/s41419-026-08648-7 (PMC13153209; doi:10.1038/s41419-026-08648-7)
Supplement: Supplementary file 5 — Supplementary Information [file 41419_2026_8648_MOESM5_ESM.pdf]

Figure 1

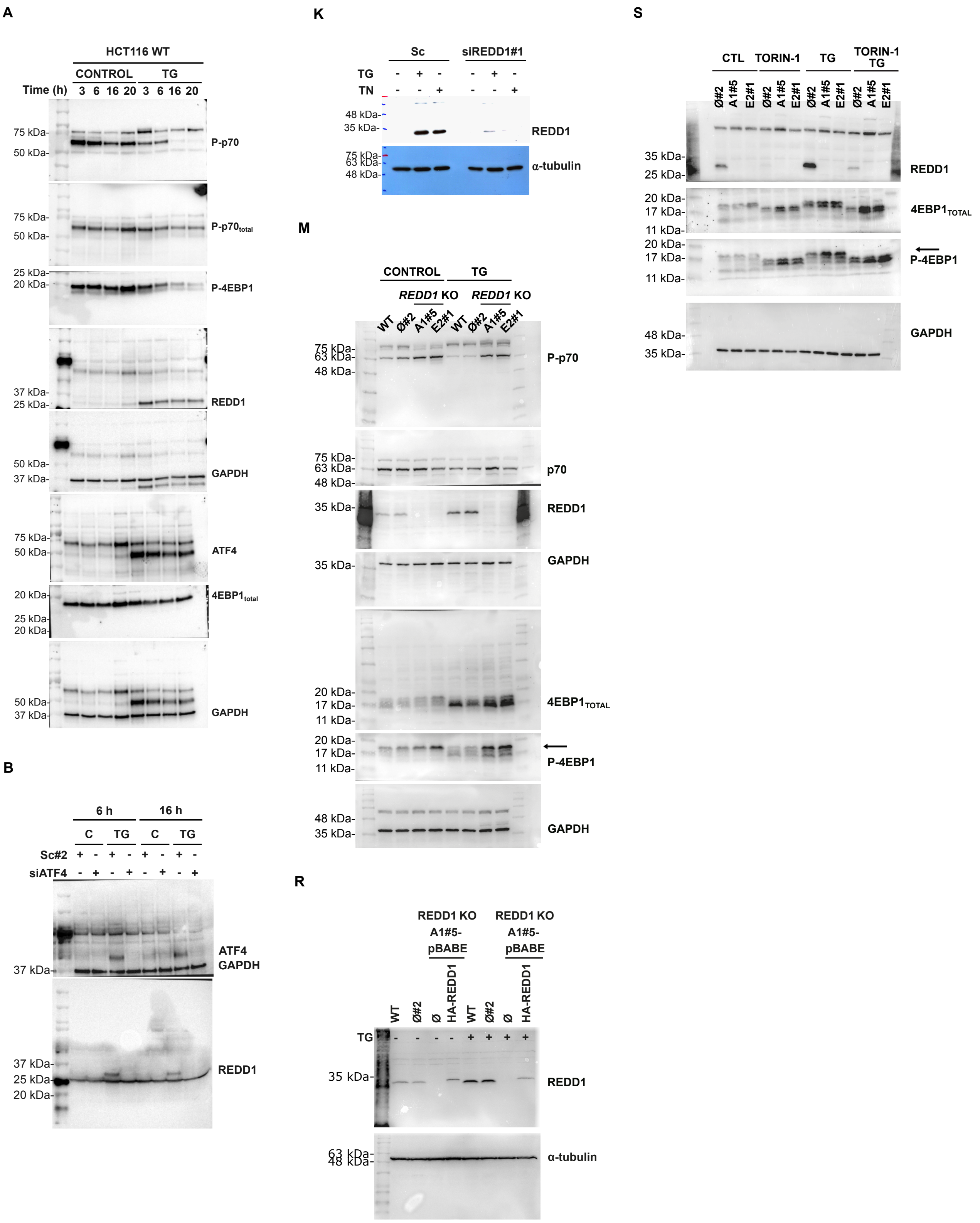

A
HCT116 WT
CONTROL
TG
Time (h) 3 6 16 20 3 6 16 20
75 kDa-
50 kDa-
P-p70
P-p70total
25 kDa-
20 kDa-
P-4EBP1
37 kDa-
25 kDa-
REDD1
50 kDa-
37 kDa-
GAPDH
75 kDa-
50 kDa-
ATF4
20 kDa-
4EBP1total
25 kDa-
20 kDa-
GAPDH
B
6 h
16 h
C TG C TG
Sc#2
siATF4
37 kDa-
ATF4
GAPDH
37 kDa-
25 kDa-
20 kDa-
REDD1
K
Sc
siREDD1#1
TG
TN
48 kDa-
35 kDa-
REDD1
75 kDa-
63 kDa-
48 kDa-
α-tubulin
M
CONTROL
TG
REDD1 KO
WT Ø#2 A1#5 E2#1
75 kDa-
63 kDa-
48 kDa-
P-p70
p70
35 kDa-
REDD1
GAPDH
20 kDa-
17 kDa-
11 kDa-
4EBP1TOTAL
P-4EBP1
48 kDa-
35 kDa-
GAPDH
R
REDD1 KO A1#5-pBABE
WT Ø#2 Ø HA-REDD1
TG
35 kDa-
REDD1
63 kDa-
48 kDa-
α-tubulin
S
CTL
TORIN-1
TG
TORIN-1 TG
Ø#2 A1#5 E2#1
35 kDa-
25 kDa-
REDD1
20 kDa-
17 kDa-
11 kDa-
4EBP1TOTAL
P-4EBP1
48 kDa-
35 kDa-
GAPDH

Figure 2

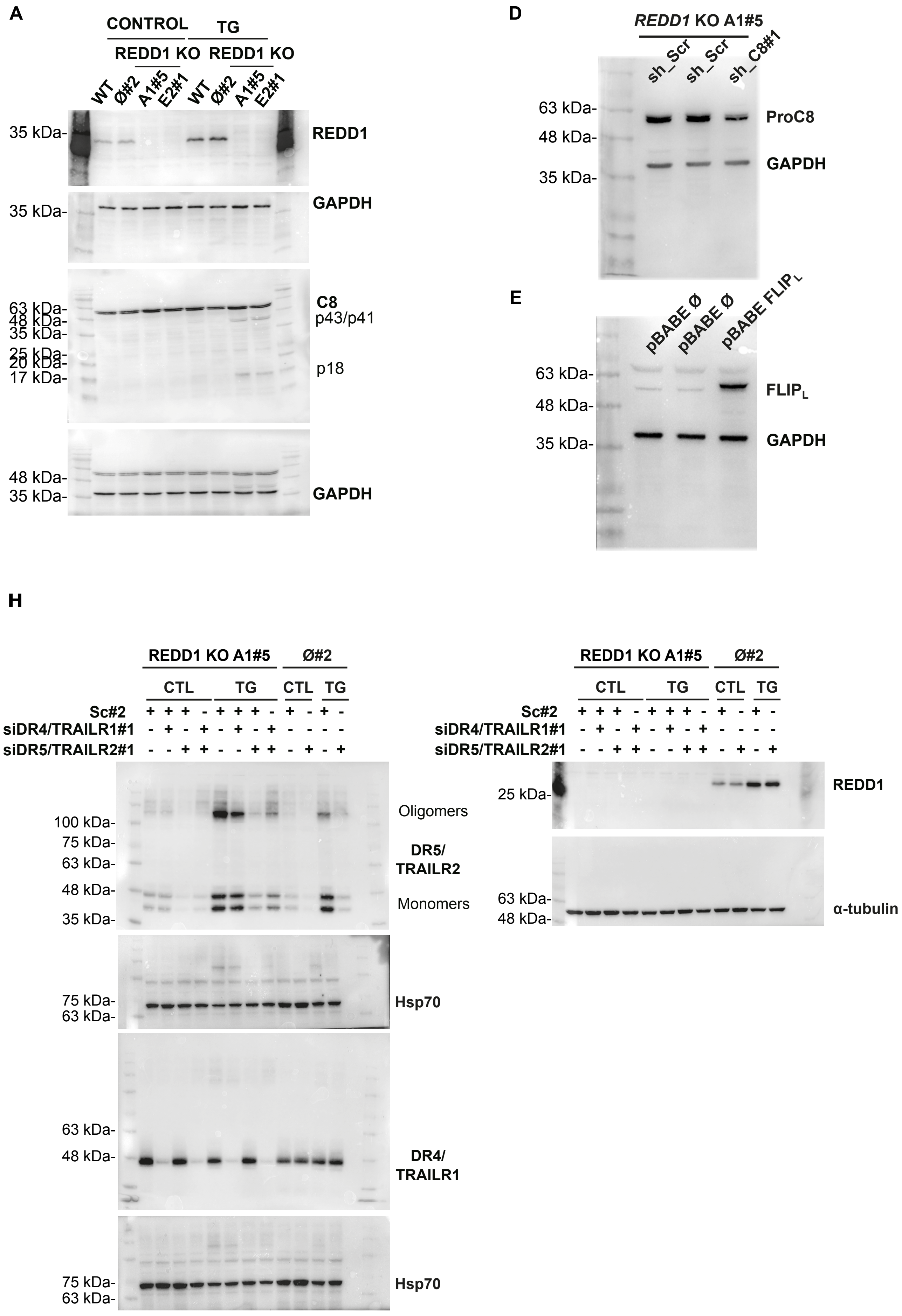
A
CONTROL
TG
REDD1 KO
REDD1 KO
WT
Ø#2
A1#5
E2#1
WT
Ø#2
A1#5
E2#1
35 kDa-
REDD1
35 kDa-
GAPDH
63 kDa-
48 kDa-
35 kDa-
25 kDa-
20 kDa-
17 kDa-
C8
p43/p41
p18
48 kDa-
35 kDa-
GAPDH
D
REDD1 KO A1#5
sh_Scr
sh_Scr
sh_C8#1
63 kDa-
48 kDa-
35 kDa-
ProC8
GAPDH
E
pBABE Ø
pBABE Ø
pBABE FLIP$_L$
63 kDa-
48 kDa-
35 kDa-
FLIP$_L$
GAPDH
H
REDD1 KO A1#5
Ø#2
CTL
TG
CTL
TG
Sc#2
siDR4/TRAILR1#1
siDR5/TRAILR2#1
100 kDa-
75 kDa-
63 kDa-
48 kDa-
35 kDa-
Oligomers
DR5/
TRAILR2
Monomers
75 kDa-
63 kDa-
Hsp70
63 kDa-
48 kDa-
DR4/
TRAILR1
75 kDa-
63 kDa-
Hsp70
25 kDa-
REDD1
63 kDa-
48 kDa-
α-tubulin

Figure 3

A

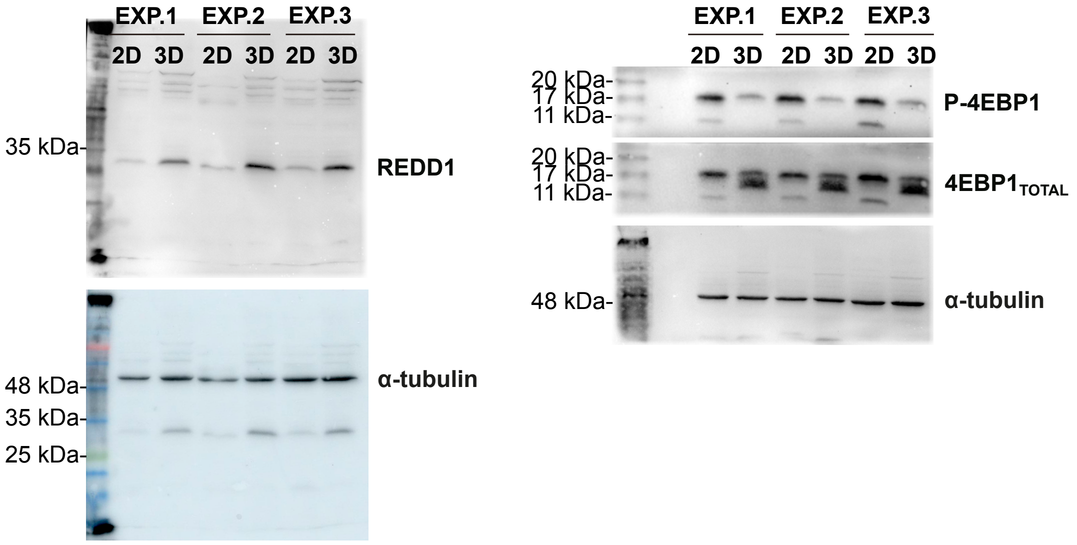

G

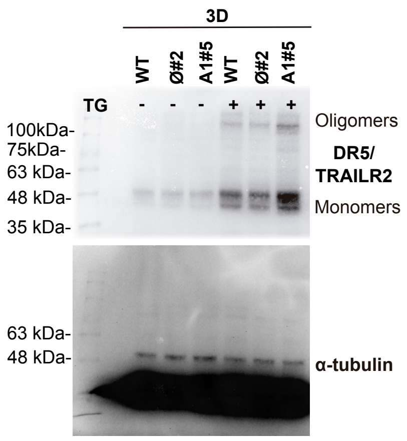

I

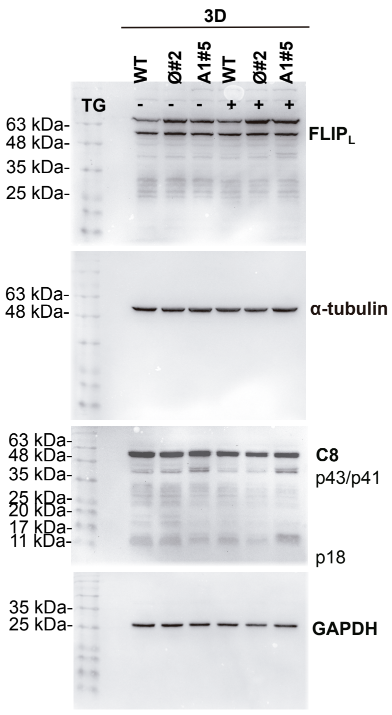

L

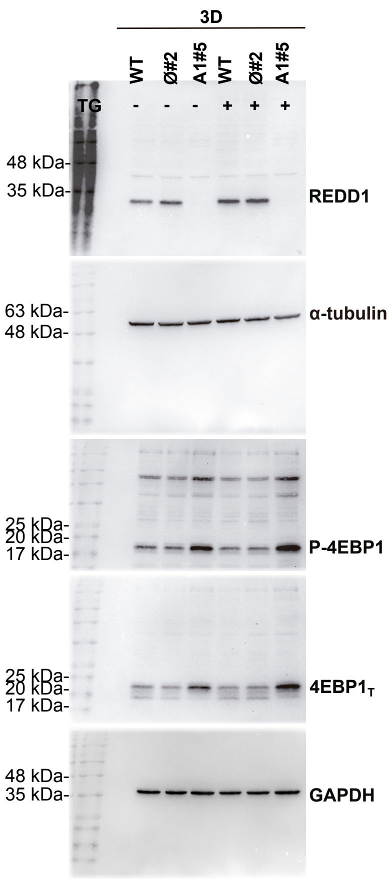

Figure 4

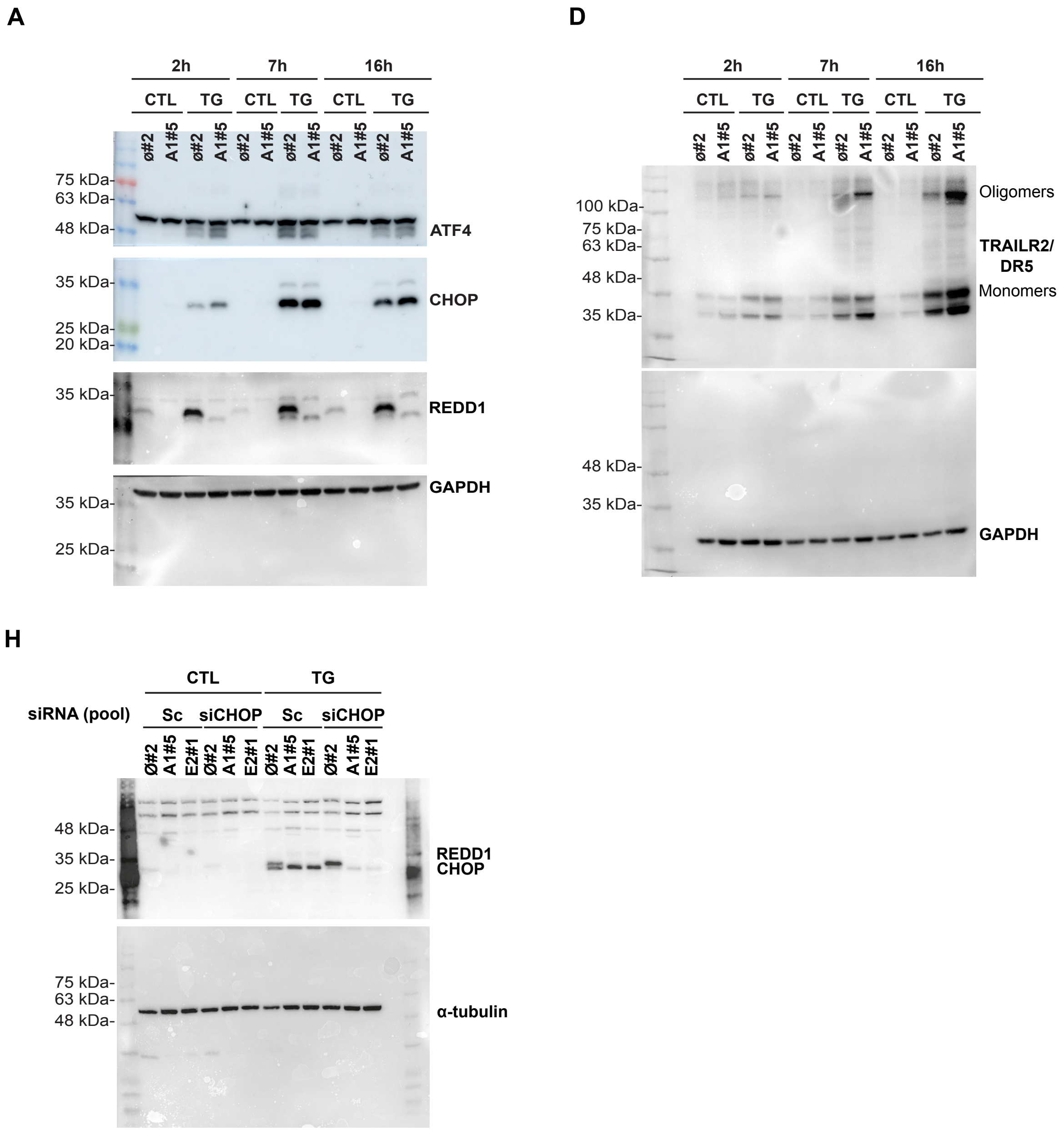
A
2h
7h
16h
CTL
TG
CTL
TG
CTL
TG
ø#2
A1#5
ø#2
A1#5
ø#2
A1#5
ø#2
A1#5
ø#2
A1#5
ø#2
A1#5
75 kDa-
63 kDa-
48 kDa-
ATF4
35 kDa-
25 kDa-
20 kDa-
CHOP
35 kDa-
REDD1
35 kDa-
25 kDa-
GAPDH
D
2h
7h
16h
CTL
TG
CTL
TG
CTL
TG
ø#2
A1#5
ø#2
A1#5
ø#2
A1#5
ø#2
A1#5
ø#2
A1#5
ø#2
A1#5
100 kDa-
75 kDa-
63 kDa-
48 kDa-
35 kDa-
Oligomers
TRAILR2/
DR5
Monomers
48 kDa-
35 kDa-
GAPDH
H
CTL
TG
siRNA (pool)
Sc
siCHOP
Sc
siCHOP
Ø#2
A1#5
E2#1
Ø#2
A1#5
E2#1
Ø#2
A1#5
E2#1
Ø#2
A1#5
E2#1
48 kDa-
35 kDa-
25 kDa-
REDD1
CHOP
75 kDa-
63 kDa-
48 kDa-
α-tubulin

Figure 5

C

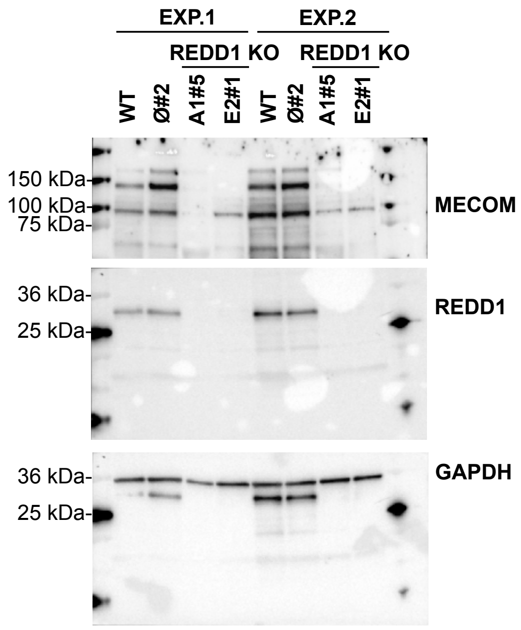

J

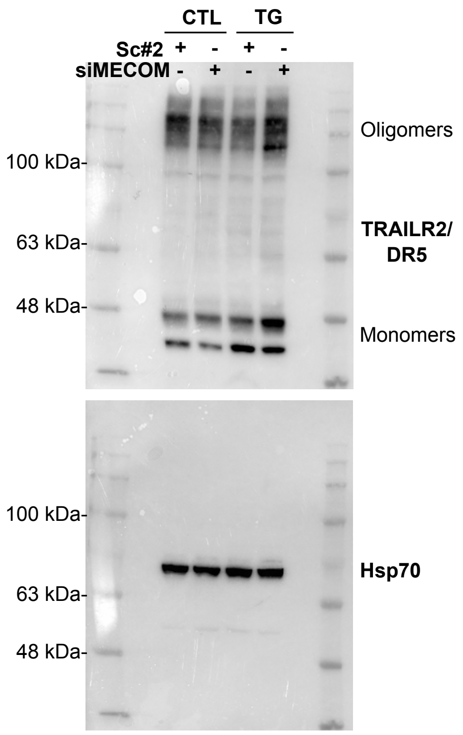

H

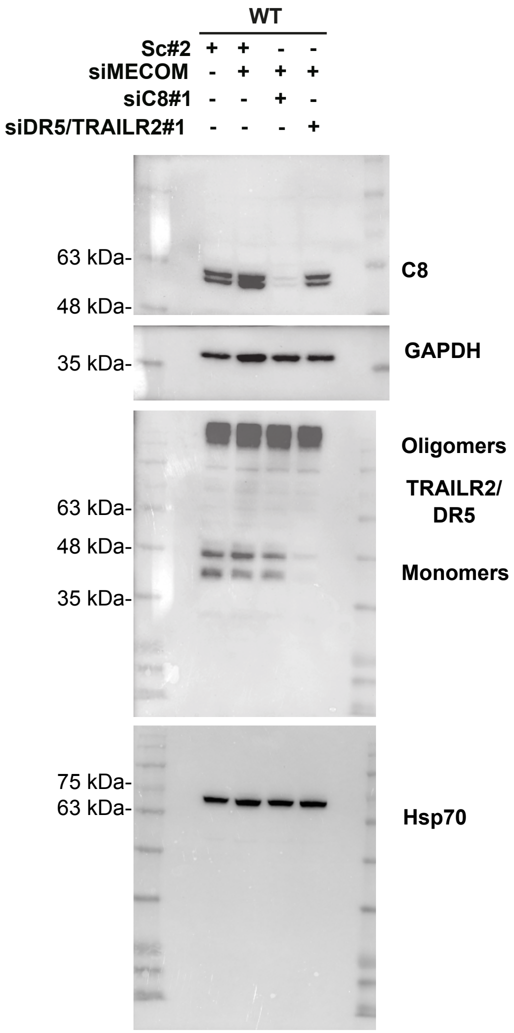

O

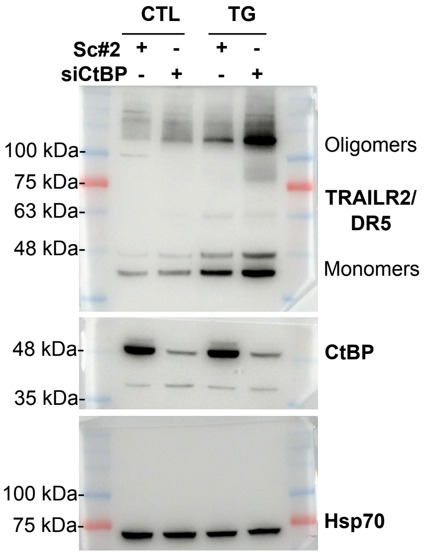

Figure S1

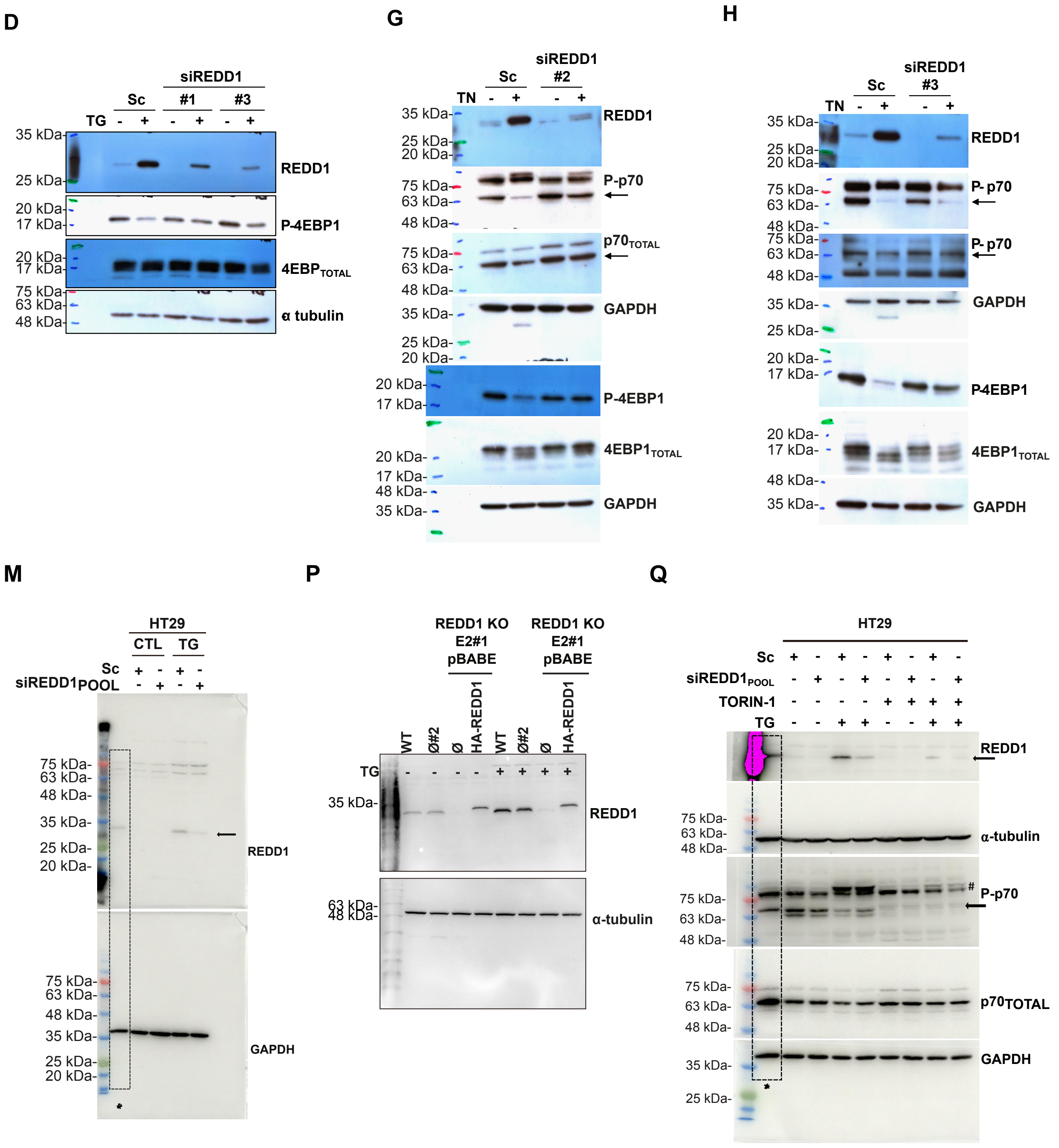

*: irrelevant sample
#: unspecific band

Figure S2

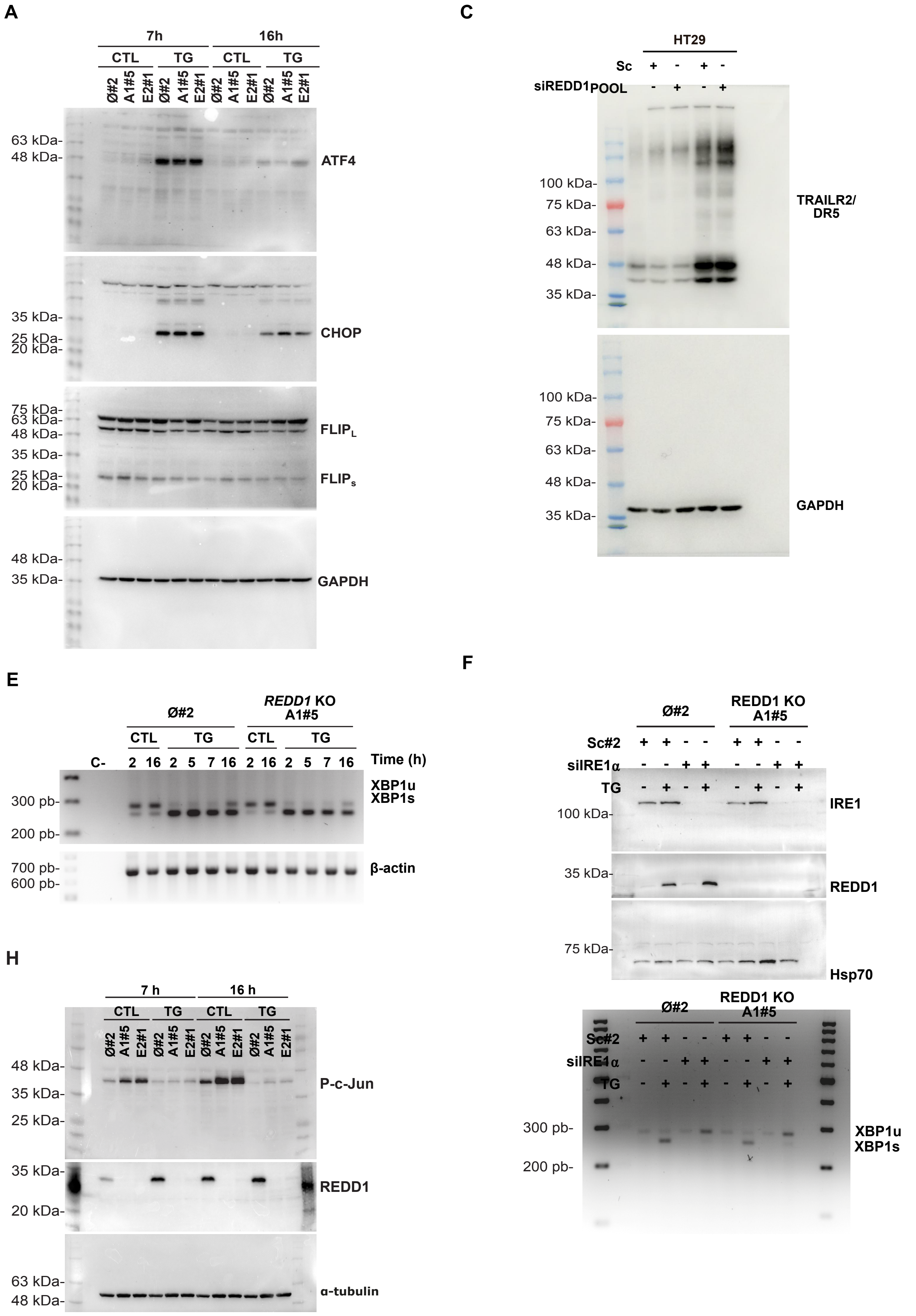

A
7h
16h
CTL
TG
CTL
TG
Ø#2
A1#5
E2#1
63 kDa-
48 kDa-
ATF4
35 kDa-
25 kDa-
20 kDa-
CHOP
75 kDa-
63 kDa-
48 kDa-
35 kDa-
25 kDa-
20 kDa-
FLIP$_L$
FLIP$_s$
48 kDa-
35 kDa-
GAPDH
C
HT29
Sc
siREDD1POOL
100 kDa-
75 kDa-
63 kDa-
48 kDa-
35 kDa-
TRAILR2/
DR5
100 kDa-
75 kDa-
63 kDa-
48 kDa-
35 kDa-
GAPDH
E
Ø#2
REDD1 KO
A1#5
CTL
TG
CTL
TG
C-
2 16 2 5 7 16 2 16 2 5 7 16
Time (h)
300 pb-
200 pb-
XBP1u
XBP1s
700 pb-
600 pb-
β-actin
F
Ø#2
REDD1 KO
A1#5
Sc#2
siIRE1α
TG
100 kDa-
IRE1
35 kDa-
REDD1
75 kDa-
Hsp70
Ø#2
REDD1 KO
A1#5
Sc#2
siIRE1α
TG
300 pb-
200 pb-
XBP1u
XBP1s
H
7 h
16 h
CTL
TG
CTL
TG
Ø#2
A1#5
E2#1
48 kDa-
35 kDa-
25 kDa-
P-c-Jun
35 kDa-
20 kDa-
REDD1
63 kDa-
48 kDa-
α-tubulin

Figure S3

B

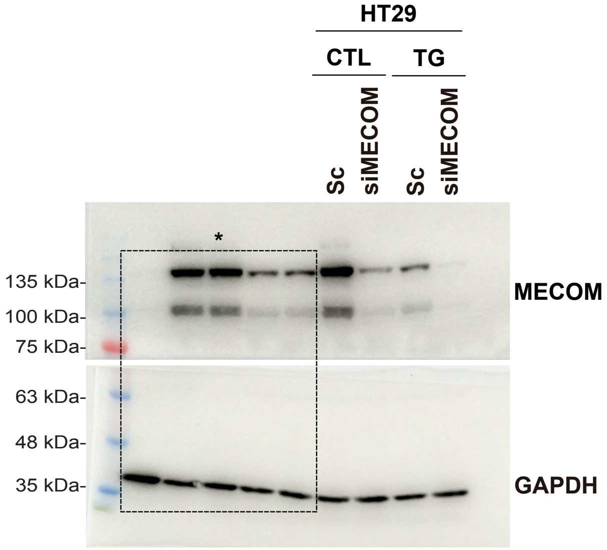

E

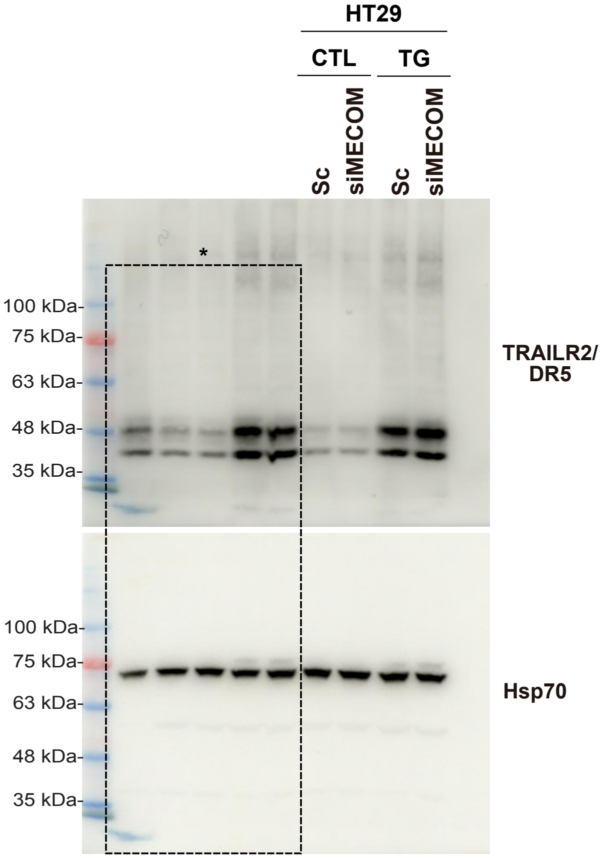

*: unreleated experiment
